# Hypoxia-responsive zinc finger E-box-binding homeobox 2 (ZEB2) regulates a network of calcium-handling genes in the injured heart

**DOI:** 10.1093/cvr/cvae163

**Published:** 2024-09-23

**Authors:** Monika M Gladka, Arwa Kohela, Anne E de Leeuw, Bas Molenaar, Danielle Versteeg, Lieneke Kooijman, Mariska van Geldorp, Willem B van Ham, Rocco Caliandro, Jody J Haigh, Toon A B van Veen, Eva van Rooij

**Affiliations:** Hubrecht Institute, Royal Netherlands Academy of Arts and Sciences (KNAW) and University Medical Centre Utrecht (UMCU), Uppsalalaan 8, 3584 CT Utrecht, The Netherlands; Department of Medical Biology, Amsterdam University Medical Center, Amsterdam Cardiovascular Sciences, Amsterdam, The Netherlands; Hubrecht Institute, Royal Netherlands Academy of Arts and Sciences (KNAW) and University Medical Centre Utrecht (UMCU), Uppsalalaan 8, 3584 CT Utrecht, The Netherlands; School of Biotechnology, Nile University, Giza, Egypt; Hubrecht Institute, Royal Netherlands Academy of Arts and Sciences (KNAW) and University Medical Centre Utrecht (UMCU), Uppsalalaan 8, 3584 CT Utrecht, The Netherlands; Hubrecht Institute, Royal Netherlands Academy of Arts and Sciences (KNAW) and University Medical Centre Utrecht (UMCU), Uppsalalaan 8, 3584 CT Utrecht, The Netherlands; Hubrecht Institute, Royal Netherlands Academy of Arts and Sciences (KNAW) and University Medical Centre Utrecht (UMCU), Uppsalalaan 8, 3584 CT Utrecht, The Netherlands; Hubrecht Institute, Royal Netherlands Academy of Arts and Sciences (KNAW) and University Medical Centre Utrecht (UMCU), Uppsalalaan 8, 3584 CT Utrecht, The Netherlands; Hubrecht Institute, Royal Netherlands Academy of Arts and Sciences (KNAW) and University Medical Centre Utrecht (UMCU), Uppsalalaan 8, 3584 CT Utrecht, The Netherlands; Department of Medical Physiology, University Medical Centre Utrecht, Utrecht, The Netherlands; Department of Medical Biology, Amsterdam University Medical Center, Amsterdam Cardiovascular Sciences, Amsterdam, The Netherlands; Department of Pharmacology and Therapeutics, University of Manitoba, Winnipeg, Canada; Department of Medical Physiology, University Medical Centre Utrecht, Utrecht, The Netherlands; Hubrecht Institute, Royal Netherlands Academy of Arts and Sciences (KNAW) and University Medical Centre Utrecht (UMCU), Uppsalalaan 8, 3584 CT Utrecht, The Netherlands; Department of Cardiology, University Medical Centre Utrecht (UMCU), Meibergdreef 9, 1105 AZ Amsterdam, The Netherlands

**Keywords:** Cardiac ischaemia, Calcium handling, Hypoxia, Transcriptional regulation, Post-transcriptional regulation, MicroRNA

## Abstract

**Aims:**

Intracellular calcium (Ca^2+^) overload is known to play a critical role in the development of cardiac dysfunction. Despite the remarkable improvement in managing the progression of heart disease, developing effective therapies for heart failure (HF) remains a challenge. A better understanding of molecular mechanisms that maintain proper Ca^2+^ levels and contractility in the injured heart could be of therapeutic value.

**Methods and results:**

Here, we report that transcription factor zinc finger E-box-binding homeobox 2 (ZEB2) is induced by hypoxia-inducible factor 1-alpha (HIF1α) in hypoxic cardiomyocytes and regulates a network of genes involved in Ca^2+^ handling and contractility during ischaemic heart disease. Gain- and loss-of-function studies in genetic mouse models revealed that ZEB2 expression in cardiomyocytes is necessary and sufficient to protect the heart against ischaemia-induced diastolic dysfunction and structural remodelling. Moreover, RNA sequencing of ZEB2-overexpressing (Zeb2 cTg) hearts post-injury implicated ZEB2 in regulating numerous Ca^2+^-handling and contractility-related genes. Mechanistically, ZEB2 overexpression increased the phosphorylation of phospholamban at both serine-16 and threonine-17, implying enhanced activity of sarcoplasmic reticulum Ca^2+^-ATPase (SERCA2a), thereby augmenting SR Ca^2+^ uptake and contractility. Furthermore, we observed a decrease in the activity of Ca^2+^-dependent calcineurin/NFAT signalling in Zeb2 cTg hearts, which is the main driver of pathological cardiac remodelling. On a post-transcriptional level, we showed that ZEB2 expression can be regulated by the cardiomyocyte-specific microRNA-208a (miR-208a). Blocking the function of miR-208a with anti-miR-208a increased ZEB2 expression in the heart and effectively protected from the development of pathological cardiac hypertrophy.

**Conclusion:**

Together, we present ZEB2 as a central regulator of contractility and Ca^2+^-handling components in the mammalian heart. Further mechanistic understanding of the role of ZEB2 in regulating Ca^2+^ homeostasis in cardiomyocytes is an essential step towards the development of improved therapies for HF.

## Graphical Abstract

**Graphical Abstract:**
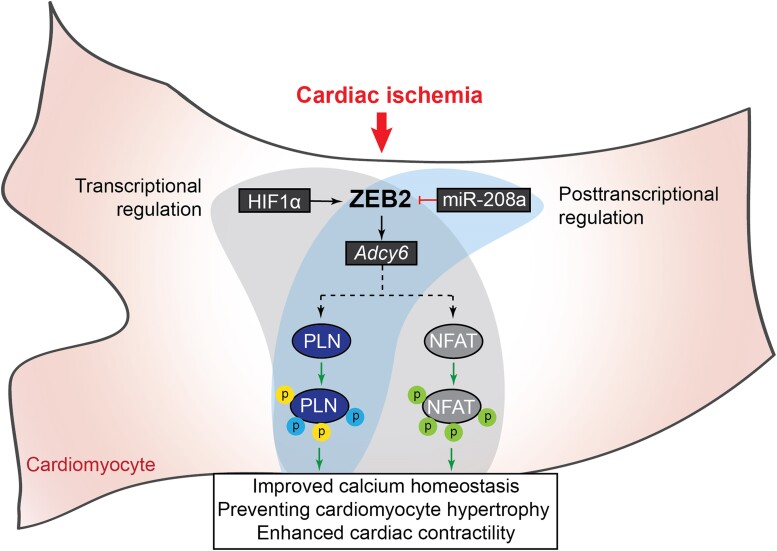

**Time of primary review: 68 days**

## 1. Introduction

Heart failure (HF) caused by ischaemic heart disease (IHD) is the most common cardiac disorder and is a leading cause of death worldwide.^1–3^ IHD is caused by the (partial) occlusion of coronary arteries, which leads to a reduction in blood flow to the heart. Current treatments for IHD include reperfusion therapy and medications that cause a significant reduction in immediate mortality rates. However, improved quality of life and long-term survival remain unattained due to the detrimental pathological remodelling accompanying IHD.^4^

A main hallmark of IHD is the hypoxia-induced loss of cardiomyocytes and the development of a rigid fibrotic scar that affects cardiac contractility.^5,6^ Within cardiomyocytes, ischaemic injury causes calcium (Ca^2+^) overload that leads to hypertrophy and perturbed contractility and eventually cardiomyocyte dysfunction.^7,8^ One major cause of this pathological remodelling is the dysregulation of a network of Ca^2+^-handling genes.^9–11^ Identification of factors which can control Ca^2+^ homeostasis after injury could contribute to the design of novel IHD therapies.

Given the well-known roles of hypoxia-inducible factor 1-alpha (HIF1α) in cardiomyocyte biology after ischaemic injury,^12–17^ we used sequencing data from the infarcted heart to identify genes downstream of HIF1α that could potentially play a role in its cardioprotective effects. In doing so, we were able to identify zinc finger E-box-binding homeobox 2 (ZEB2) as a downstream transcription factor of HIF1α in cardiomyocytes, which is induced during hypoxia and decreases in expression with age. ZEB2 is a central regulator of epithelial-to-mesenchymal transition (EMT),^18,19^ cellular dedifferentiation, and foetal development.^20,21^ Recently, we further identified ZEB2 as a key cardioprotective factor, which signals to endothelial cells in a cell non-autonomous manner to promote angiogenesis, hence maintaining cardiac function after ischaemic damage.^22,23^

Here, we show that upon ischaemic injury, an increase in ZEB2 in cardiomyocytes induces a gene network involved in cardiac contraction and Ca^2+^ handling, which corresponds to a better maintenance in function after ischaemic damage. We further show that ZEB2 is post-transcriptionally regulated by miR-208a and that the therapeutic inhibition of miR-208a can effectively increase ZEB2 levels and prevent pathological remodelling and cardiac dysfunction. Our findings demonstrated that under hypoxic conditions, ZEB2 controls a transcriptional network of Ca^2+^-handling genes in cardiomyocytes, which subsequently mediates the contractile function of the heart.

## 2. Methods

### 2.1 Mice

Animal studies were performed according to the guidelines from Directive 2010/63/EU of the European Parliament on the protection of animals used for scientific purposes. Animal experiments were approved by the institutional policies and regulations of the Animal Welfare Committee of the Royal Netherlands Academy of Arts and Sciences (HI 13.2304, AVD8011002015250 16.2305/IVD366) and following the Guidelines for the Care and Use of Laboratory Animals. Mice were housed with 12:12 h light:dark cycle in a temperature-controlled room with access to food and water *ad libitum*. We used 8–9-week-old male and female mice for all animal experiments as indicated. The number of mice used represents the minimum required to achieve statistical significance based on previous experience and power calculations. Mice were randomly allocated into experimental groups, and the investigator was blinded to the experimental group if possible.

### 2.2 Human heart samples

Approval for studies on human tissue samples was obtained from the Medical Ethics Committee of the University Medical Center Utrecht, The Netherlands (12#387). Written informed consent was obtained or, in certain cases, waived by the ethics committee when obtaining informed consent was not possible due to the death of the patient. None of the co-authors was involved in tissue collection. Tissue samples were anonymized before the access was obtained. In this study, we included tissue from the left ventricular (LV)–free wall of patients with end-stage HF secondary to IHD. The end-stage HF tissue was obtained during heart transplantation or at autopsy. Correlation analysis for *ZEB2* and *HIF1α* was performed on tissue samples from the LV-free wall of control hearts and patients with IHD (ischaemic region, border zone, and remote region).

### 2.3 Transgenic mouse models

Mice used in this study were generated as previously described.^22^ Rosa26-LoxStopLoxZeb2 mice^24^ were crossed with mice harbouring a Cre recombinase under the control of the murine *Myh6* promoter (αMHC-Cre Tg mice),^25^ to generate αMHC-Cre R26-lslZeb2/lslZeb2 (*Zeb2* cTg) mice. Mice harbouring a floxed allele of *Zeb2* (*Zeb2* fl/fl*)*^26^ were crossed to αMHC-Cre Tg mice to generate Cre-*Zeb2* fl/fl (*Zeb2* cKO) mice. Mice were housed in normal conditions with 12:12 h light:dark cycles in a temperature-controlled room with food and water *ad libitum*. For all animal experiments, we used 8–9-week-old male mice. All mice were genotyped by polymerase chain reaction (PCR) using primers shown in Supplementary material online, *Table S4*. The sample size was determined by a power calculation based on an echocardiographic effect size. Biotechnicians were blinded to group allocation during the experiment and when assessing the outcome.

### 2.4 Ischaemia–reperfusion

Ischaemia–reperfusion (IR) was performed by temporary (1 h) ligation of the left anterior descending artery (LAD). Mice were injected subcutaneously with buprenorphine (0.05–0.1 mg/kg) as an analgesic at least 30 min prior to surgery to alleviate pain or distress. When multiple surgeries take place on the same day, all animals received buprenorphine at the same time in the morning. After 30 min (or longer), mice were anaesthetized with a mix of fentanyl (0.05 mg/kg), midazolam (5 mg/kg), and dexmedetomidine (0.125 mg/kg) via intraperitoneal injection and supplemented with 1–2% isoflurane to maintain a surgical plane of anaesthesia. Immediately after the surgery, anaesthesia was reversed using atipamezole. Mice received a second subcutaneous injection of buprenorphine (0.05–0.1 mg/kg) 8–12 h after the first dose to provide additional pain relief. The third dose of buprenorphine (0.05–0.1 mg/kg) was subcutaneously administered approximately 12 h later (the following day after surgery). After the anaesthesia, mice were intubated, and the tracheal tube was connected to a ventilator (UNO Micro Ventilator UMV-03). Hair was removed from the thorax and neck, and the surgical site was cleaned with iodine and 70% ethanol. Skin was subsequently incised at the midline to allow access to the left third intercostal space. Pectoral muscles were retracted, and the intercostal muscles were cut caudal to the third rib. Wound hooks were placed to allow access to the heart. The pericardium was incised longitudinally, and a 7.0 silk suture was placed around the LAD and a piece of 2–3 mm polyethylene (PE) 10 tubing. One hour later, the PE tubing was removed, and the ligature was cut to allow for reperfusion. Following the surgery, the rib cage was closed with a 5.0 silk suture, and the skin was closed with a wound clip. Mice were disconnected from the ventilator by removing the tracheal tube and placed on a nose cone with 100% oxygen. During the whole procedure and recovery period, animals were placed on a 38°C heating mat.

### 2.5 Echocardiography

Cardiac function was evaluated by two-dimensional transthoracic echocardiography on sedated mice (2% isoflurane) using a Visual Sonic Ultrasound system with a 30 MHz transducer (VisualSonics Inc., Toronto, Canada). Hearts were imaged in a parasternal long-axis and short-axis view at the level of the papillary muscles to record M-mode measurements and determine heart rate, wall thickness, and end-diastolic and end-systolic dimensions. Cardiac contractile function was assessed by fractional shortening (FS) (defined as the end-diastolic dimension minus the end-systolic dimension normalized for the end-diastolic dimension) and ejection fraction (EF) (defined as the stroke volume normalized for the end-diastolic volume).

### 2.6 Euthanasia and tissue collection

After echocardiography, mice were sacrificed by an overdose of isoflurane (3–4%), followed by cervical dislocation for tissue collection.

### 2.7 Tomo-seq

Tomo-seq experiments were performed as described previously.^27^ In short, heart samples were embedded in tissue freezing medium, frozen on dry ice, and cryosectioned into 48 slices of 80 µm thickness. Next, ribonucleic acid (RNA) was extracted from individual slices, and Illumina sequencing libraries were barcoded according to the CEL-seq protocol.^28^ Paired-end reads obtained by Illumina sequencing were aligned to the transcriptome using BWA.^29^ The 5′ mate of each pair was used for mapping, discarding all reads that mapped equally well to multiple loci. The 3′ mate was used for barcode information. Read counts were first normalized to total counts per section and then renormalized to the median of total reads across sections in order to ensure that count numbers roughly corresponded to the number of mapped reads. Tomo-seq data analysis was performed in MATLAB (MathWorks) using custom-written code. An expression cut-off of >4 reads in >1 section was used.

### 2.8 Isolation of ventricular cardiomyocytes from neonatal rats

Neonatal rat cardiomyocytes (NRCM) were isolated by enzymatic dissociation of 1–2-day-old neonatal rat hearts. In brief, pups were placed on ice for 5–10 min for light anaesthesia. After decapitation, hearts were collected, and ventricles were separated from atria and cut into small pieces in a balanced salt solution prior to enzymatic digestion using trypsin (Thermo Fisher Scientific, #15400054) under constant stirring at 37°C. The supernatant, containing intact cardiomyocytes, was collected, centrifuged at 1500 rpm for 4 min, and resuspended in Ham F10 medium (Thermo Fisher Scientific, #11550043) supplemented with 5% FBS, 10% L-glutamine, and 10% Pen-Strep. Collected cells were seeded onto uncoated 100 mm plastic dishes for 1.5 h at 37°C in 5% CO_2_ humidified atmosphere. Subsequently, the supernatant, which consists mainly of non-adhering cardiomyocytes, was collected, and cells were counted and plated on gelatine-coated 6-well plates 1 × 10^6^ cells per well. After 24 h, the medium was changed to Ham F10 supplemented with insulin–transferrin–sodium selenite supplement (Roche), 10% L-glutamine, and 10% Pen-Strep. Cells were used for the hypoxia study, small interfering RNA (siRNA)–mediated knock-downs and infection with AAV9 virus as described below.

### 2.9 Hypoxia study

NRCMs were isolated and 1 × 10^6^ cells were plated per well in 6-well plates. For hypoxia treatment, cells were placed in a hypoxia chamber with 1% O_2_ and 5% CO_2_ and incubated for 1, 2, 4, 6, 8, or 24 h. For normoxia treatment, control cells were placed for 24 h in a regular cell culture incubator. RNA was isolated after each time point as described below. For immunohistochemistry, cells were cultured for 6 h under normoxic or hypoxic conditions, fixed, and stained as described below.

### 2.10 Promoter analysis

We used rVista 2.0 (https://rvista.dcode.org) to compare the *Zeb2* and *Adcy6* promoter regions between mouse and human and illustrated our data as percentage of conservation of a 10.0 kb genomic region upstream of transcriptional start site of *Zeb2* or *Adcy6*.

### 2.11 Measurements and analysis of Ca^2+^ transients

Cardiomyocytes were isolated as described before.^30^ Cells were subsequently incubated in 1:1000 Ca^2+^-sensitive dye Fluo-4-AM (Thermo Fisher, #F14201) in Tyrode solution containing (mM) NaCl (130), KCl (4), CaCl_2_ (1.8), MgCl_2_ (1.2), NaHCO_3_ (18), HEPES (10), and glucose (10), for 15 min at 37 degrees. Cells were placed in Tyrode solution during the recording of the Ca^2+^ transients and were paced at 1, 3, and 5 Hz by field stimulation. Recordings were made on a custom-built microscope (Cairn Research, Kent, UK) using a 10× objective. Blue light was used for excitation, using a 482/35 excitation filter (Semrock FF01-482/35-25) and captured using a 514 long-pass emission filter (Semrock LP02-514RU-25), with a high-speed camera (Andor Zyla 5.5.CL3, Oxford Instruments). Analysis was performed using a custom MATLAB script (https://osf.io/86ufe/). Fluorescence signals were normalized to their own baseline intensity to allow for comparisons between conditions. Ca^2+^ traces were selected from the data set, and signal noise was filtered to visualize representative Ca^2+^ transients.

### 2.12 RNA isolation and quantitative real-time PCR

RNA was isolated using TRIzol reagent (Invitrogen) and reverse transcribed into complementary DNA (cDNA) using iScript cDNA Synthesis Kit (Bio-Rad, #1708891) according to the manufacturer’s instructions. Quantitative real-time PCR (qPCR) was performed using iQ SYBR Green Supermix (Bio-Rad, #170-8885) on the CFX96 Real-Time PCR instrument (Bio-Rad). Transcript levels were normalized for endogenous loading. Primer sequences are provided in Supplementary material online, *Table S5*.

### 2.13 RNA Sequencing

Total RNA was extracted from remote zones of hearts using TRIzol reagent (Invitrogen). RNA sequencing (RNA-seq) libraries were prepared using the TruSeq Stranded Total RNA Library Prep Kit (Illumina) with Invitrogen according to the manufacturer’s instructions. Next, strand-specific single-end 75 bp reads were generated on an Illumina NextSeq 500. Reads were aligned and quantified against the Gencode.M4 gtf list for annotated genes using the STAR workflow. Heart libraries were sequenced with a minimum of 14 million reads [16.2 ± 1.9 (mean ± SD)]. Differential expression was analysed using DESeq v1.2211 using per condition dispersion estimates.

### 2.14 Gene ontology and pathway analysis

To identify whether gene groups shared similar biological functions, differentially expressed gene groups were analysed using Kyoto Encyclopedia of Genes and Genomes pathway and Gene Ontology biological processe database using DAVID7. Significant enrichment of genes was shown, and *P* values were corrected for multiple testing using the Benjamini–Hochberg method.

### 2.15 Western blot analysis

Heart tissue lysates were collected in RIPA buffer [50 mM Tris-HCl pH 7.5, 150 mM NaCl, 0.1% SDS, 0.5% sodium deoxycholate (Sigma-Aldrich), 1% Triton X-100 (Sigma-Aldrich), protease inhibitor (Roche)], and protein concentration was determined using a Bradford assay (Bio-Rad). Samples were boiled in 4× Laemmli buffer, including 2% β-mercaptoethanol for 5 min at 99°C. SDS-PAGE and Western Blot were performed using Mini-PROTEAN Tetra Vertical Electrophoresis Cell with Mini Trans-Blot (Bio-Rad). Membranes were blocked in 3–5% non-fat dry milk and incubated overnight at 4°C with primary antibodies (see Supplementary material online, *Table S6*). On the next day, blots were incubated with the corresponding peroxidase-conjugated AffiniPure secondary antibodies (Jackson ImmunoResearch) for 45 min and proteins were visualized using ECL solution (Bio-Rad, #170-5061) on the ImageQuant LAS4000 imaging system. Western blots were quantified using Fiji software.

### 2.16 Immunofluorescence staining

Immunofluorescence imaging was performed on paraffin-embedded heart sections and fixed *in vitro* cultures. Paraffin-embedded heart sections were deparaffinized and re-hydrated in an alcohol gradient. Sections were subsequently boiled in boiling ethylenediaminetetraacetic acid buffer pH 9 for 20 min for antigen retrieval, blocked with 0.05% BSA, and incubated with primary antibodies (see Supplementary material online, *Table S5*) overnight at 4°C. On the next day, sections were washed and incubated with the corresponding Alexa Fluor secondary antibodies (Thermo Fisher Scientific) for 1 h followed by 4′,6-diamidino-2-phenylindole (DAPI) 1:5000 (Invitrogen, #D3571) for 10 min at room temperature (RT). Sections were finally mounted with ProLong Gold Antifade Mountant (Invitrogen, #P36934) for imaging. Fluorescein isothiocyanate–labelled wheat germ agglutinin (Sigma-Aldrich, #L4895) was used to visualize and quantify cardiomyocyte cross-sectional area with ImageJ software. For *in vitro* cultures, cells were fixed with 4% paraformaldehyde, quenched with NH4Cl, permeabilized, blocked with 1% fish gelatine (gelatine from cold-water fish skin, Sigma-Aldrich, #G7765), and incubated with primary antibodies (see Supplementary material online, *Table S5*) for 25 min at RT. Cells were then incubated with the corresponding Alexa Fluor secondary antibodies (Thermo Fisher Scientific) for 20 min at RT. Cells were finally washed and sealed with mounting medium (ProLong Gold Antifade Mountant with DAPI, Invitrogen, #P36935). Imaging was performed using the Leica TCS SPE confocal microscope.

### 2.17 siRNA experiments

siRNA trilencers purchased from OriGene were used to knock down ZEB2 (#SR511798), HIF1α (#SR510711), and AC6 (#SR511901). A scrambled siRNA was used as a non-targeting control (#SR30002). Knock-down was performed at a final concentration of 10 nM using Lipofectamine 2000 (Thermo Fisher Scientific, #11668027) for 24 h. Next, medium was refreshed for an additional 8 h, and cells were harvested for analysis. Cells were subjected to hypoxia (1% O_2_ and 5% CO_2_) for 6 h before collection.

### 2.18 Human heart samples

Approval for studies on human tissue samples was obtained from the Medical Ethics Committee of the University Medical Center Utrecht, The Netherlands (12#387). Written informed consent was obtained or in certain cases waived by the ethics committee when obtaining informed consent was not possible due to death of the patient. In this study, we used tissue from the LV-free wall of patients with end-stage HF secondary to IHD. Tissue was obtained during heart transplantation or upon autopsy. RNA was isolated as previously described, and gene expression values obtained by qPCR were plotted for correlation analysis.

### 2.19 MicroRNA target prediction

For the identification of putative microRNAs targeting Zeb2, we used the target prediction tools miRBase (http://www.mirbase.org) and TargetScan (http://www.targetscan.org/vert_72/).

### 2.20 Luciferase assay

HEK293T cells were transfected using Lipofectamine (Thermo Fisher Scientific, #11668027) with pMIR-reporter plasmid containing the 3′ untranslated region (3′UTR) of ZEB2 (25 ng/well), pCMV plasmid containing miR-208a (at different concentrations), and *Renilla*. After 48 h, luciferase activity was measured using the Dual-Luciferase® Reporter Assay System (Promega). Relative luciferase activity was normalized to *Renilla* expression.

### 2.21 Anti-miR injection in mice

For the baseline study, adult C56BL/6J mice (Charles Rivers) were injected with anti-miR-control or anti-miR-208a (25 mg/kg) subcutaneously for three consecutive days. Animals were sacrificed 3, 7, or 14 days after the last injection, and cardiac tissues were collected for baseline analysis. For the IR study, adult mice received sham or IR surgery which was followed by subcutaneous anti-miR injections for three consecutive days and used for functional and molecular analysis 14 days later.

### 2.22 Statistical and reproducibility

The number of samples (*n*) used in each experiment is shown in the figures and indicates biological replicates. Results are presented as the mean ± standard error of the mean (SEM). Statistical analyses were performed using PRISM (GraphPad Software Inc. version 6). Two groups were statistically compared using Student’s *t*-test. Multiple groups were statistically compared using ordinary one-way ANOVA or two-way ANOVA. Outliers were defined by Grubbs’ test (alpha = 0.05). Data are represented as mean ± SEM. Differences were considered statistically significant at *P* < 0.05. In the figures, asterisks indicate statistical significance (*P < 0.05, **P < 0.01, ***P < 0.001, ****P < 0.0001) which is also indicated in the individual figures. All representative images of the hearts or cells were selected from at least three independent experiments with similar results, unless indicated differently in the figure legend.

## 3. Results

### 3.1 *Hif1α* induces *Zeb2* expression in hypoxic cardiomyocytes

**Figure 1:**
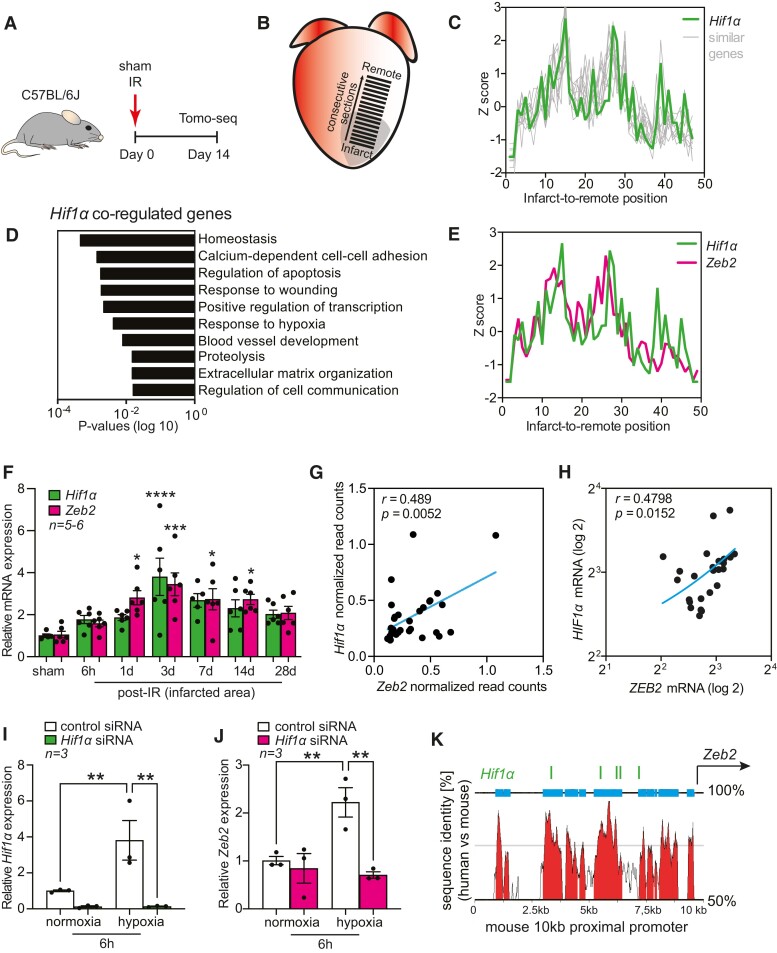
Zeb2 is induced by HIF1α in hypoxic cardiomyocytes. (*A*) Study design. (*B*) Tomo-seq on infarcted mouse heart. (*C*) Spatial expression traces of *Hif1α* and similarly regulated genes in mouse hearts 14 days post-injury. (*D*) Gene Ontology analysis showing enriched pathways of *Hif1α* co-regulated genes. (*E*) Spatial expression traces of *Hif1α* and *Zeb2* in the infarcted mouse heart. (*F*) qPCR analysis of *Hif1α* and *Zeb2* expression levels in mouse hearts collected at different time points after IR. (*G–H*) Pearson correlation between *Hif1α* and Zeb2 expression in (*G*) single cardiomyocytes isolated from injured mouse hearts and (*H*) human ischaemic hearts. (*I* and *J*) qPCR analysis of (*I*) *Hif1α* and (*J*) *Zeb2* expression levels following *Hif1α* knock-down in NRCMs. (*K*) UCSC Genome Browser annotation of the 10 kb proximal promoter region of *Zeb2* showing multiple conserved HREs. *n* (biological replicates) is indicated in the figures. Data are represented as mean ± SEM, *P < 0.05, **P < 0.01, and ***P < 0.001 compared to control (corresponding sham or normoxia) using one-way ANOVA followed by Dunnett’s multiple comparison test (*F*) or unpaired, two-tailed Student’s *t*-test (*I* and *J*). NRCMs, neonatal rat cardiomyocytes; HRE, hypoxia-responsive elements.

In an effort to define novel mechanisms that underlie cardiac remodelling after ischaemic injury, we generated a spatial gene expression atlas of the infarcted mouse heart using Tomo-seq.^27,31^ By sequencing consecutive sections, we were able to obtain local gene expression profiles spanning from the infarct to the remote area (*Figure 1A* and *B*). Local expression cues enabled us to trace the expression of critical factors involved in specific aspects of cardiac remodelling. Considering the importance of *Hif1α* during cardiac ischaemia, we screened for the top 100 genes that showed the highest correlation with *Hif1α* expression across the infarcted heart (*Figure 1C*, Supplementary material online, *Figure S1A* and *B*). As expected, these genes were functionally linked to response to wounding and hypoxia (*Figure 1D*), confirming a link between the genes and *Hif1α*. Next to known *Hif1α* interactors, such as *Egr1*, *Adam10,* and *Abcg2*,^32–34^ we identified *Zeb2* as one of the top *Hif1α* co-regulated transcription factors (*Figure 1E*). By qPCR analysis, we could further validate a strong correlation between *Hif1α* and *Zeb2* expression in infarcted areas of hypoxic hearts at several time points after injury (*Figure 1F*). A positive correlation was also observed in single cardiomyocytes isolated from injured mouse hearts (*Figure 1G*) and in human ischaemic hearts (*Figure 1H*). To address the possible regulation of *Zeb2* by HIF1α in cardiomyocytes, we performed siRNA-mediated knock-down of endogenous *Hif1α* in primary NRCM subjected to normoxia or hypoxia for 6 h (see Supplementary material online, *Figure S2A*). This led to a reduction in the expression of *Hif1α* (*Figure 1I*), known as HIF1α target genes (see Supplementary material online, *Figure S2B* and *C*), and a decrease in *Zeb2* transcript levels (*Figure 1J*). When examining the *Zeb2* promoter region, we identified five conserved hypoxia-responsive elements (HREs), which could potentially explain the observed HIF1α-dependent *Zeb2* expression (*Figure 1K*, Supplementary material online, *Figure S2D–F*).

**Figure 2:**
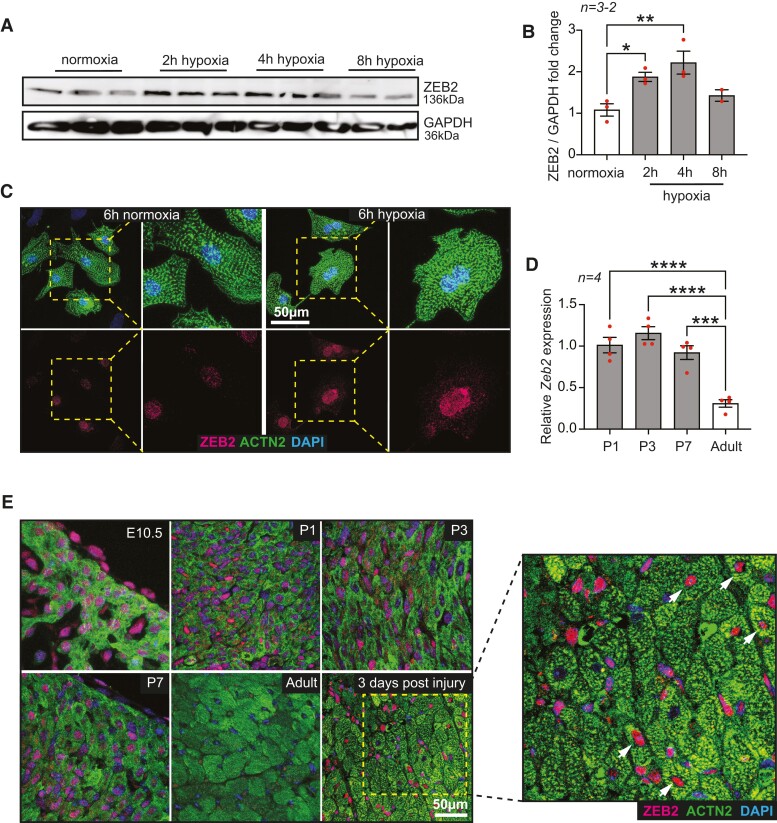
ZEB2 expression is induced in response to hypoxia. (*A*) Western blot analysis of ZEB2 and (*B*) the quantification in NRCMs subjected to normoxia and hypoxia for indicated time points. (*C*) Representative immunofluorescence staining of ZEB2 and ACTN2 in normoxic and hypoxic NRCMs. Insets show magnified regions. (*D*) qPCR expression analysis of Zeb2 at different developmental stages of mouse hearts. (*E*) Representative immunofluorescence staining of ZEB2 and ACTN2 in mouse hearts at different developmental time points and after ischaemic injury. *n* (biological replicates) is indicated in the figures. White arrows show ZEB2-positive cardiomyocytes. Data are represented as mean ± SEM, *P < 0.05, **P < 0.01, ***P < 0.001, and ****P < 0.0001 compared to control (normoxia or adult heart) using one-way ANOVA followed by Dunnett’s multiple comparison test (*B* and *D*).

Western Blot analysis shows increased ZEB2 expression in NRCMs subjected to hypoxia compared to normoxia for indicated time points (*Figure 2A* and *B*). Immunofluorescent staining of ZEB2 in NRCMs showed an induction of ZEB2 protein after 6 h of hypoxia (*Figure 2C*). Since embryonic and neonatal hearts are more hypoxic than adult hearts,^35^ we also checked the expression of *Zeb2* during different developmental stages. High expression levels of *Zeb2* were observed in embryonic and postnatal mouse hearts on mRNA (*Figure 2D*) and protein (*Figure 2E*) levels, followed by a significant decrease in adult hearts and an induction 3 days after ischaemic injury (*Figure 2D*). Altogether, these data strongly suggest a HIF1α-mediated *Zeb2* induction in cardiomyocytes during hypoxia.

### 3.2 Cardiomyocyte-specific ZEB2 overexpression protects from IR-induced pathological hypertrophy and contractile dysfunction

**Figure 3:**
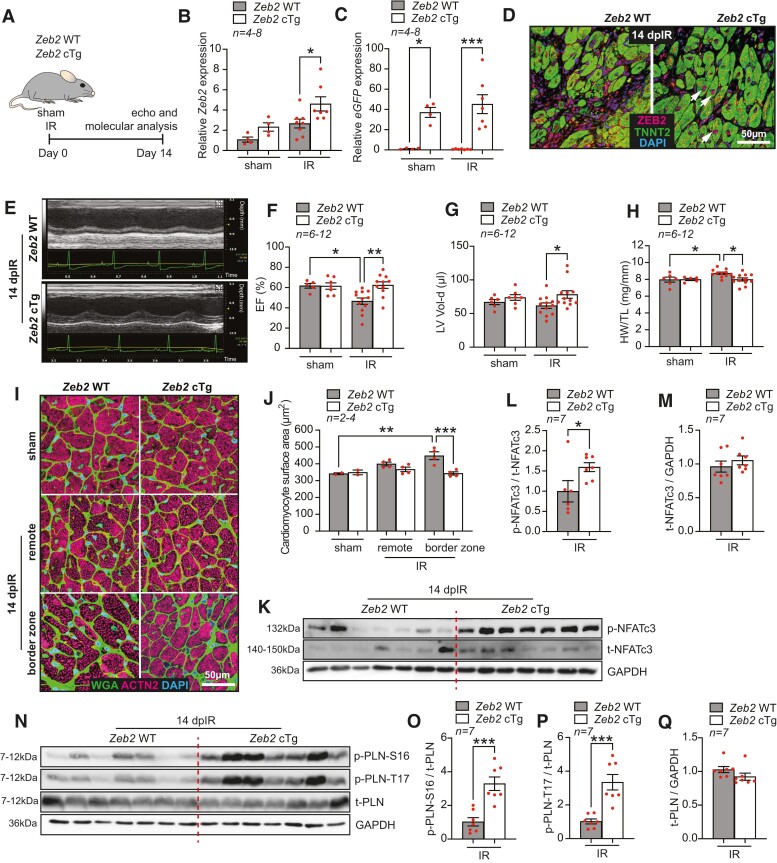
Cardiomyocyte-specific ZEB2 overexpression protects from ischaemia-induced pathological hypertrophy and contractile dysfunction. (*A*) Study design. (*B* and *C*) qPCR analysis of (*B*) Zeb2 and (*C*) eGFP (Ct values) in the hearts from Zeb2 WT and Zeb2 cTg mice post sham or IR surgeries. (*D*) Representative immunofluorescence staining of ZEB2 and TNNT2 in the hearts from Zeb2 WT and Zeb2 cTg mice 14 days post-IR (dpIR). (*E*) Representative M-mode images of Zeb2 WT and Zeb2 cTg mice 14 dpIR. (*F–H*) Quantification of (*F*) ejection fraction (EF), (*G*) left ventricular volume in diastole (LV Vol-d), and (*H*) heart weight to tibia length (HW/TL) ratio in Zeb2 WT and Zeb2 cTg mice post-surgery. (*I*) WGA staining to show cardiomyocyte surface area and (*J*) its quantification. (*K–Q*) Western blot analysis of (*K* and *N*) the indicated proteins and (*L*, *M*, and *O–Q*) their quantification in Zeb2 WT and Zeb2 cTg mice 14 dpIR. *n* (biological replicates) is indicated in the figures. White arrows show ZEB2-positive cardiomyocytes. Data are represented as mean ± SEM, *P < 0.05, **P < 0.01, and ***P < 0.001 using one-way ANOVA followed by Sidak’s multiple comparison test (*B*, *C*, *F*, *G*, and *H*), compared to sham using one-way ANOVA followed by Dunnett’s multiple comparison test (*J*) or compared to Zeb2 WT using unpaired, two-tailed Student’s *t*-test (*L*, *M*, *O*, *P*, and *Q*).

To study the *in vivo* effects of ZEB2 induction in cardiomyocytes, we generated cardiomyocyte-specific ZEB2-overexpressing mice (Zeb2 cTg) as described previously^22^ and subjected them to IR injury. Functional and molecular analysis 14 days after surgery confirmed the upregulation of ZEB2 at mRNA and protein levels in Zeb2 cTg mice compared to their wild-type (WT) littermates (Zeb2 WT) (*Figure 3A–D*). Echocardiographical measurements showed an improvement in function and a better maintained cardiac morphology in Zeb2 cTg mice (*Figure 3E–G*, Supplementary material online, *Figure S3A–C* and *Table S1*). Additionally, heart weight to tibia length (HW/TL) ratio indicated a reduction in pathological hypertrophy in Zeb2 cTg mice post-IR compared to WT controls (*Figure 3H*). This was confirmed by a reduction in the surface area of cardiomyocytes located at border zone and remote areas of the injured hearts (*Figure 3I* and *J*).

Calcineurin is a phosphatase that upon increased levels of cytosolic Ca^2+^ dephosphorylates nuclear factor of activated T cells (NFAT), which in turn translocates to the nucleus to activate a hypertrophic gene programme.^36,37^ While several NFAT isoforms have been detected in the heart,^38,39^ NFATc3 plays a dominant role in cardiac hypertrophic signalling.^40^ In line with the pro-hypertrophic role of the calcineurin/NFAT signalling pathway, we observed significantly higher levels of NFATc3 phosphorylation (p-NFATc3) in Zeb2 cTg post-IR hearts compared to the Zeb2 WT group with no increase in total NFATc3 (t-NFATc3) (*Figure 3K–M*), implying less calcineurin activity upon increased ZEB2 levels. This effect was not observed when comparing the sham groups of both genotypes (see Supplementary material online, *Figure S3D–F*).

Since calcineurin is activated during an intracellular increase in Ca^2+^,^41,42^ we next examined whether the Ca^2+^-handling machinery was affected in Zeb2-overexpressing hearts. Phospholamban (PLN) is a key regulator of cardiac contractility that modulates Ca^2+^ sequestration in the sarcoplasmic reticulum (SR) via sarco/endoplasmic reticulum Ca^2+^ (SERCA2a). Phosphorylation of PLN relieves the inhibitory effect of PLN on SERCA2a, which leads to a faster relaxation and an increase in contraction.^43,44^ This can occur either through beta-adrenergic stimulation and enhanced cyclic adenosine monophosphate (cAMP)–dependent protein kinase A activity at serine-16 (S16) or the activation of the Ca^2+^/calmodulin-dependent CamKII at threonine-17 (T17).^45,46^ Cardiomyocyte-specific overexpression of ZEB2 increased PLN phosphorylation at both S16 and T17 (*Figure 3N–Q*), likely contributing to the enhanced cardiac contractility observed in these mice after injury. This increase in phosphorylation of PLN was also seen, although not significantly, in sham hearts from Zeb2 cTg mice, further exemplifying the role of ZEB2 in mediating these effects (see Supplementary material online, *Figure S3G–J*). Together, these data indicate that ZEB2 overexpression in cardiomyocytes prevents cardiac dysfunction and cardiomyocyte hypertrophy post-ischaemic injury.

### 3.3 Cardiac overexpression of ZEB2 alters the expression of Ca^2+^-handling genes in the heart

**Figure 4:**
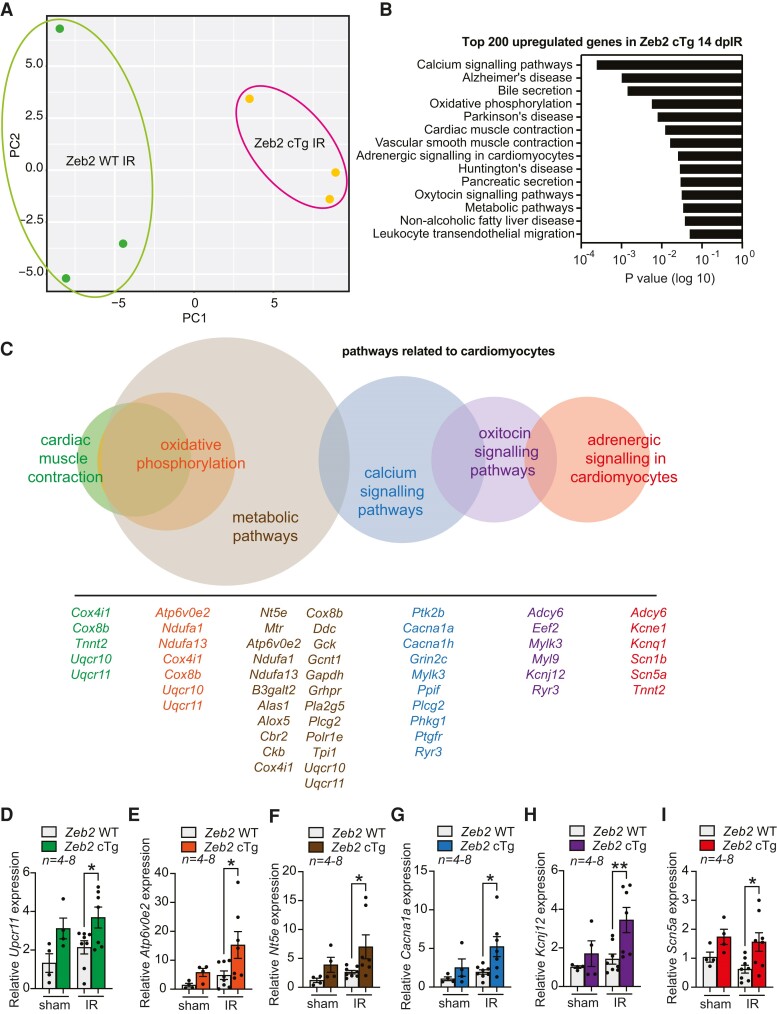
RNA-seq analysis on the hearts from injured Zeb2 WT and Zeb2 cTg mice reveals altered expression of Ca^2+^-handling genes. (*A*) Principal component analysis (PCA) showing the distribution of RNA-seq data transcripts from Zeb2 WT and Zeb2 cTg mice 14 days post-IR (dpIR). (*B*) Gene ontology analysis showing enriched pathways of the top 200 upregulated genes on RNA-seq in Zeb2 cTg vs. Zeb2 WT mice 14 dpIR. (*C*) Multi-set Venn diagram illustrating pathway Gene Ontology analysis related to cardiomyocyte function of the top upregulated genes on RNA-seq in Zeb2 cTg vs. Zeb2 WT mice 14 dpIR. (*D–I*) qPCR analysis of the indicated genes representative of the different pathways in *C* in Zeb2 cTg vs. Zeb2 WT mice 14 days post sham or IR. *n* (biological replicates) is indicated in the figures. Data are represented as mean ± SEM, *P < 0.05, and **P < 0.01 compared to Zeb2 WT using Ordinary one-way ANOVA followed by Sidak’s multiple comparison test (*D*, *E*, *F*, *G*, *H*, *I*, and *J*).

To examine the role of ZEB2 in injured cardiomyocytes in more detail, we performed RNA-seq on cardiac tissue from Zeb2 WT and Zeb2 cTg mice subjected to IR (*Figure 4A*). Pathway analysis of the top 200 significantly upregulated genes was linked to Ca^2+^ signalling and cardiac muscle contraction, further suggesting a role for ZEB2 in regulating these processes (*Figure 4B* and *C*). The expression of the top upregulated genes was confirmed by qPCR in a larger sample set (*n* = 7–8) (*Figure 4D–I*).

To address the consequences of the observed increase in expression of cardiac contractility-related genes, we used our existing RNA-seq data of WT sham vs. WT IR hearts to check how expression of these genes is altered upon injury. We observed that the expression of various genes related to cardiomyocyte contractility is reduced upon ischaemic injury (see Supplementary material online, *Figure S4A*), which correlates with a decline in cardiac function.^47–51^ This decrease was restored upon ZEB2 overexpression in the ischaemic heart (see Supplementary material online, *Figure S4B*), suggesting a ZEB2-mediated rescue of contractility-related genes after ischaemic injury.

Among the top upregulated genes in Zeb2 cTg hearts post-injury, we identified adenylate cyclase 6 (*Adcy6*) (see Supplementary material online, *Figure S4A–C*). ADCY6 is a membrane-associated enzyme that catalyses the formation of cAMP. cAMP is crucial for intracellular signal transduction pathways and is responsible for phosphorylation of several proteins important in Ca^2+^ homeostasis and cardiac contraction.^52^ To determine whether ZEB2 can transcriptionally regulate ADCY6, we analysed the 10 kb proximal promoter region of *Adcy6*, by which we identified three conserved ZEB2 binding motifs (see Supplementary material online, *Figure S4D* and *E*). Additionally, we made use of existing ChIP-seq data (https://www.encodeproject.org/targets/ZEB2-human/) and confirmed that these ZEB2 binding motifs are located in enhancer regions of *Adcy6* (see Supplementary material online, *Figure S4F* and *G*). These data indicate that ZEB2 could play a role in the transcriptional regulation of ADCY6 in the heart, potentially contributing to the cardioprotective effects of ZEB2 overexpression after injury.

### 3.4 ZEB2 improves Ca^2+^ handling in cardiomyocytes after injury

**Figure 5:**
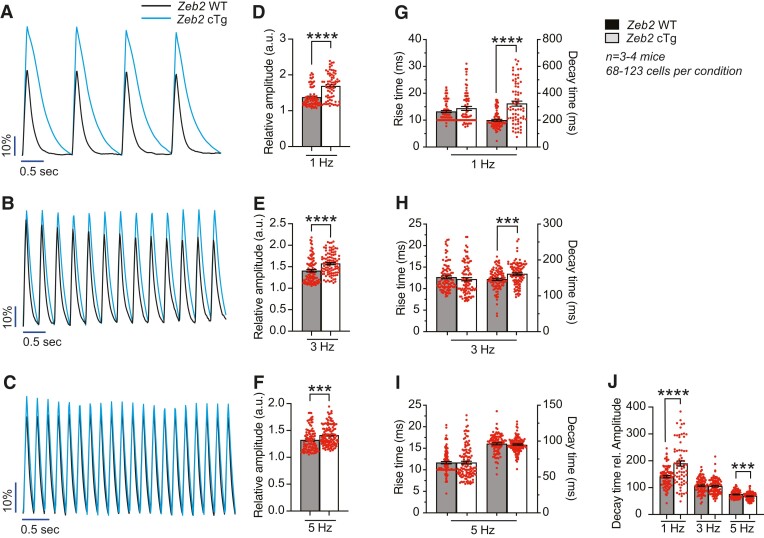
ZEB2 overexpression improves Ca^2+^ handling in cardiomyocytes after injury. (*A–C*) Representative analysis of Ca^2+^ transients in cardiomyocytes isolated from Zeb2 WT and Zeb2 cTg mice 14 days post-IR (dpIR) exposed to a Ca^2+^-sensitive dye after different stimulation frequencies. (*D–F*) Quantification of relative Ca^2+^ transient amplitude. (*G–I*) Quantification of rise time, defined as the time from baseline to transient peak, and decay time, defined as the time from the transient peak back to baseline. (*J*) Quantification of decay time related to amplitude. *n* (biological replicates) is indicated in the figures. Data are represented as mean ± SEM, ***P < 0.001, and ****P < 0.0001 compared to Zeb2 WT using unpaired, two-tailed Student’s *t*-test (*D*, *E*, *F*, *G*, *H*, *I*, and *J*).

Given the observed changes in contractility and Ca^2+^-handling genes, we next measured Ca^2+^ levels in adult cardiomyocytes isolated from Zeb2 WT and Zeb2 cTg mice post-IR. Isolated cardiomyocytes were incubated with the Ca^2+^-sensitive dye Fluo-4-AM, after which changes in signal intensity, indicative of Ca^2+^ transients, were measured at different pacing frequencies (1, 3, and 5 Hz) (*Figure 5A–C*). Zeb2-overexpressing cardiomyocytes showed increased transient amplitudes at all frequencies (*Figure 5D–F*). While no differences were seen in rising time, longer decay times were observed in cells from Zeb2 cTg mice at 1 and 3 Hz (*Figure 5G–I*). Interestingly, when correcting the decay time for the total amount of Ca^2+^ represented by the amplitude, Ca^2+^ is removed more efficiently from the cytosol at a physiological pacing frequency of 5 Hz, as seen by a shorter corrected decay time (*Figure 5J*). These data demonstrate that cardiomyocytes isolated from Zeb2 cTg hearts cycle an increased amount of Ca^2+^ and have improved Ca^2+^ reuptake after IR injury, which can potentially contribute to the enhanced cardiac contractility and relaxation measured *in vivo*.

### 3.5 Genetic deletion of ZEB2 from cardiomyocytes causes a mild decline of cardiac function post-ischaemic injury but does not alter Ca^2+^ signalling

To investigate whether ZEB2 deletion would negatively impact cardiac Ca^2+^ handling, we generated cardiomyocyte-specific Zeb2 knockout mice (Zeb2 cKO), as described previously,^22^ and subjected them to IR injury (see Supplementary material online, *Figure S5A*). Loss of ZEB2 from cardiomyocytes was confirmed by qPCR and immunofluorescence (see Supplementary material online, *Figure S5B* and *C*) and did not affect the gross morphology and function of the heart at baseline.^22^ However, after IR, Zeb2 cKO mice displayed a decrease in EF and FS compared to Zeb2 fl/fl WT littermates (see Supplementary material online, *Figure S5D–G* and *Table S2*), indicating augmented cardiac remodelling in the absence of ZEB2. While a trending increase was observed for cardiomyocytes’ surface area and levels of phosphorylated NFATc3 and PLN (see Supplementary material online, *Figure S5H–Q*), this did not reach statistical significance. Additionally, *Adcy6* expression remained unchanged in Zeb2 cKO post-injury (see Supplementary material online, *Figure S5R*). Taken together, these data indicate that ZEB2 deletion from cardiomyocytes does not have a pronounced effect on cardiac function and Ca^2+^ handling 14 days post-IR.

### 3.6 *Zeb2* is post-transcriptionally regulated by microRNA-208a

Based on the cardioprotective effects of ZEB2 in cardiomyocytes, we aimed to explore ways to increase cardiac *Zeb2* expression. As microRNAs are known to function as post-transcriptional regulators, we screened for microRNAs that could potentially target *Zeb2*. Using miRBase and TargetScan as target prediction tools, we identified two potential binding sites for *microRNA-208a* (*miR-208a*) in the *Zeb2* 3′UTR (see Supplementary material online, *Figure S6A*). *miR-208a* is located within an intron of the α-myosin heavy chain (αMHC) gene, making it the sole cardiomyocyte-specific microRNA.^53^ When looking at the expression of both transcripts in the IR timeline, we observed an inverted correlation between *Zeb2* and *miR-208a*, indicating a possible regulation of *Zeb2* by *miR-208a* (see Supplementary material online, *Figure S6B*). To confirm this regulation, we generated a luciferase reporter carrying the sequence of the *Zeb2* 3′UTR in which we mutated the predicted first, second, or both seed regions of *miR-208a* (see Supplementary material online, *Figure S6C*). Dose-dependent overexpression of *miR-208a* in human embryonic kidney (HEK-293) cells resulted in a lowering of the luciferase reporter activity (see Supplementary material online, *Figure S6D* and *E*), indicating direct binding between *miR-208a* and *Zeb2.* This interaction was lost when the first or both binding sides were mutated, but no effects were observed when we only disrupted the second binding site (see Supplementary material online, *Figure S6E*), indicating that *miR-208a* is able to bind and regulate *Zeb2* via the first binding site.

To evaluate if this interaction also takes place *in vivo*, we used anti-miRs to target and inhibit *miR-208a* expression in the heart.^54^ Anti-miRs are a class of chemically engineered oligonucleotides perfectly complementary to the selected microRNA and are therefore used for silencing purposes. C57BL/6J mice were injected with anti-miR-208a (anti-208a) or anti-miR-control (anti-control) (25 mg/kg) for three consecutive days, and tissue was collected for molecular analysis 3, 7, or 14 days after the first injection (see Supplementary material online, *Figure S6F*). We were able to efficiently inhibit *miR-208a* levels in the heart at the different time points, while no significant effects on *Zeb2* expression were observed (see Supplementary material online, *Figure S6G*). Additionally, anti-miR-208a treatment did not induce any gross morphological and histological changes, suggesting that anti-miR targeting of *Zeb2* was not effective under baseline conditions (see Supplementary material online, *Figure S6H–K*).

### 3.7 miR-208a inhibition increases ZEB2 levels and improves cardiac function post-IR

**Figure 6:**
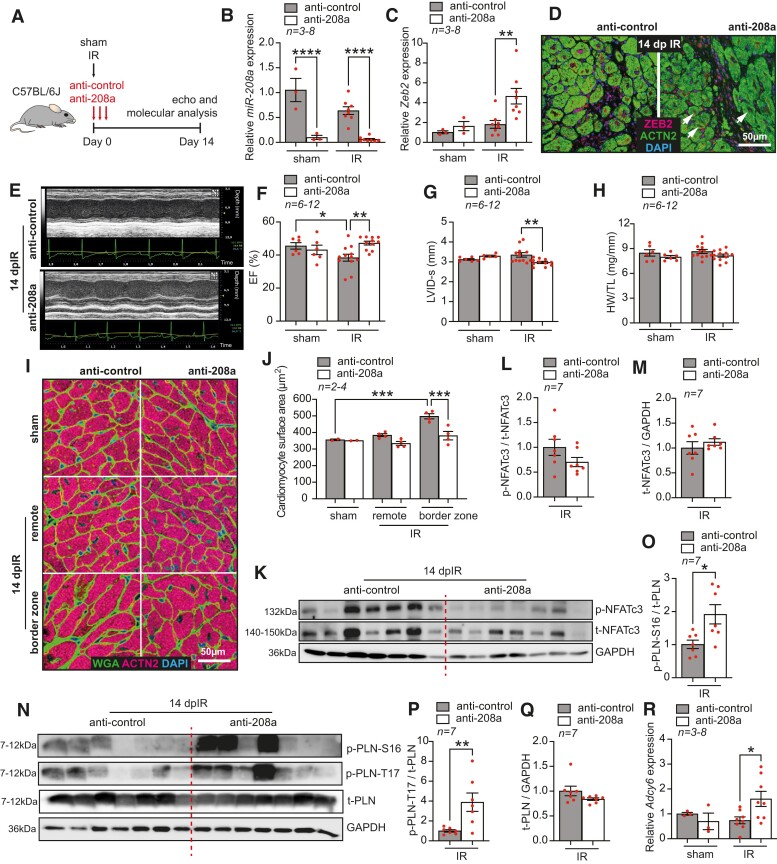
miR-208a inhibition increases ZEB2 levels and improves cardiac function post-injury. (*A*) Study design. (*B–C*) qPCR analysis of (*B*) miR-208a and (*C*) Zeb2 in WT mouse hearts treated with anti-control or anti-208a subjected to sham or IR surgeries. (*D*) Representative immunofluorescence staining of ZEB2 and ACTN2 in mouse hearts treated with anti-control or anti-208a 14 days post-IR (dpIR). (*E*) Representative M-mode images of hearts from mice treated with anti-control or anti-208a 14 dpIR. (*F–H*) Quantification of (*F*) ejection fraction (EF), (*G*) left ventricular internal diameter in systole (LVID-s), and (*H*) heart weight to tibia length (HW/TL) ratio in mice treated with anti-control or anti-208a. (*I*) WGA staining to show cardiomyocyte surface area and (*J*) its quantification. (*K–Q*) Western blot analysis of (*K* and *N*) the indicated proteins and (*L*, *M*, and *O*–*Q*) their quantification in hearts from WT mice treated with anti-control or anti-208a 14 dpIR. (*R*) qPCR analysis of *Adcy6* expression in anti-control or anti-208a-treated mice 14 days post-surgery. *n* (biological replicates) is indicated in the figures. White arrows show ZEB2-positive cardiomyocytes. Data are represented as mean ± SEM, *P < 0.05, **P < 0.01, and ***P < 0.001 using one-way ANOVA followed by Sidak’s multiple comparison test (*B*, *C*, *F*, *G*, *H*, and *R*), compared to sham using one-way ANOVA followed by Dunnett’s multiple comparison test (*J*) or compared to anti-208a-treated IR group using unpaired, two-tailed Student’s *t*-test (*L*, *M*, *O*, *P*, *Q*, *S*, and *T*).

Since stress conditions are known to alter the biogenesis and function of microRNAs,^55,56^ we next investigated whether the therapeutic inhibition of *miR-208a* would increase the expression of *Zeb2* after ischaemic injury. To do so, we subjected adult C57BL/6J mice to sham or IR surgeries followed by the systemic delivery of anti-control or anti-208a (25 mg/kg) for three consecutive days. Functional and molecular analyses were performed 14 days after the first injection (*Figure 6A*). We observed that anti-208a treatment efficiently inhibited *miR-208a*, which consequently increased *Zeb2* expression (*Figure 6B–D*). This correlated with a rescue from cardiac dysfunction (*Figure 6E–G*, Supplementary material online, *Table S3*) and cardiomyocyte hypertrophy post-IR (*Figure 6H–J*). In contrast to Zeb2 cTg mice, we did not observe an increase in phosphorylated NFATc3 levels after anti-208a treatment (*Figure 6K–M*), indicating that *miR-208a* regulates the hypertrophic response in an NFAT-independent manner.^54^ However, similar to Zeb2 cTg, anti-208a-treated mice showed an increase in phosphorylated forms of PLN (*Figure 6N–Q*) and in the expression of *Adcy6* mRNA (*Figure 6R*).

**Figure 7:**
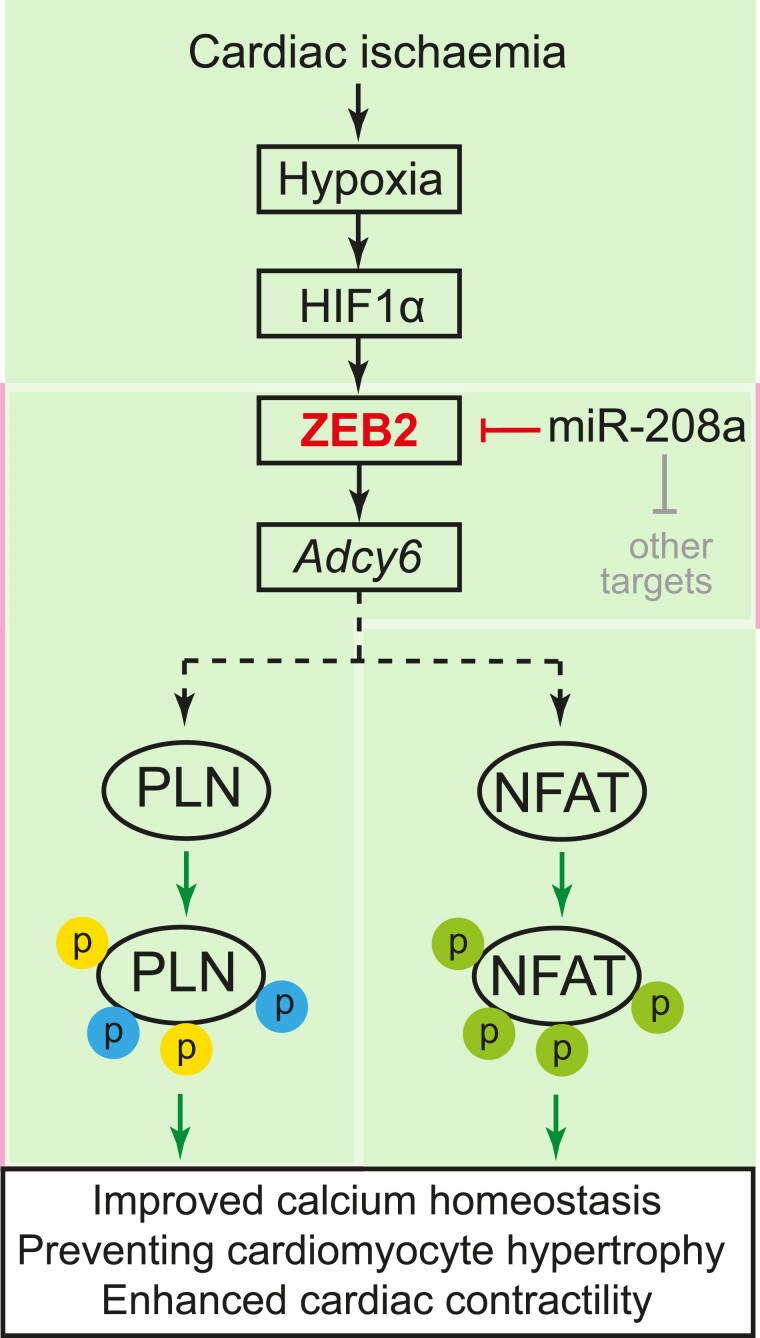
Model depicting the function of ZEB2 in the ischaemic heart. Upon cardiac ischaemia, ZEB2 is transcriptionally regulated by HIF1α and post-transcriptionally by miR-208a, resulting in transcriptional activation of Adcy6, which will trigger cardioprotective signalling by improving Ca^2+^ homeostasis, preventing cardiomyocyte hypertrophy and enhancing cardiac contractility.

Altogether, our data show that ZEB2 is regulated transcriptionally by HIF1α and post-transcriptionally by *miR-208a* in the ischaemic heart. An increase in ZEB2 expression in cardiomyocytes improves Ca^2+^ homeostasis, reduces hypertrophy, and prevents cardiac dysfunction in an Adcy6-mediated mechanism (*Figure 7*).

## 4. Discussion

In this study, we describe a key role for transcription factor ZEB2 as a downstream effector of HIF1α signalling that regulates cardiac Ca^2+^ handling and remodelling during ischaemic injury. Using Tomo-seq, we identified *ZEB2* as a direct HIF1α target gene in the ischaemic heart (*Figure 1*). Transgenic and therapeutic induction of ZEB2 expression in cardiomyocytes resulted in an improvement of contractility which coincided with a reduction in hypertrophic remodelling in response to stress (*Figures 3* and *6*). These data point to a cardioprotective role of ZEB2 via the regulation of key Ca^2+^ handling and hypertrophic signalling pathways within the cardiomyocyte.

Maintaining the right and flexible balance between oxygen demand and availability requires a tight regulation on the molecular level.^57^ Hypoxia-inducible factors 1 (HIF1) proteins are the central regulators of hypoxia responses. However, only HIF1α is affected by oxygen levels as it is rapidly degraded under homeostatic conditions. In response to hypoxia, HIF1α is stabilized and is translocated to the nucleus where it binds to HRE on DNA and activates the transcription of hypoxia-responsive genes.^58^ Studies using loss- and gain-of-function models have underlined the importance of HIF1α in the heart. Targeted HIF1α inactivation in mice has been reported to cause embryonic lethality due to abnormal vascular development and hypertrophy of the developing heart.^59,60^ This has been further supported by the altered vascularization, energy availability, Ca^2+^ flux, and contractility following cardiomyocyte-specific HIF1α deletion.^61^ On the contrary, several reports showed that the prolonged induction of HIF1α increases the mechanical load on the heart and leads to HF.^62–64^ Further studies were also able to link hypoxia and HIF1α to cardiac regeneration.^14–17^ This has mainly been attributed to a reduction in mitochondrial reactive oxygen species,^65^ decreased oxidative DNA damage, and cardiomyocyte proliferation.^14,17^ The role of HIF1α in the heart is therefore broad, which we aimed to understand better by analysing its downstream effectors their cardioprotective potential. While the HIF1α/ZEB2 axis was previously described in podocytes and in cells undergoing EMT,^66,67^ this is the first study reporting the relevance of this interaction in cardiac biology (see Supplementary material online, *Figure S2*).

Ca^2+^ is a key signalling molecule in cardiomyocytes, regulating both cardiomyocyte function and morphology. Under baseline conditions, intracellular levels of Ca^2+^ are tightly regulated through the preservation of a Ca^2+^ gradient across endoplasmic or sarcoplasmic reticulum membranes created by Ca^2+^ channels, ATPase pumps, transporters, and exchangers operating in synergy with Ca^2+^-binding proteins.^50^ In cardiomyocytes, a balanced flow of Ca^2+^ into and out of the cell controls excitation-contraction (EC) coupling^68^ and hence cardiac contraction (systole) and relaxation (diastole).

During HF, one of the most profound cellular changes is an increase in systolic Ca^2+^ levels and prolongation of the Ca^2+^ transient during diastole, leading to defects in EC coupling, cardiac dysfunction, and HF.^50^ EC coupling is regulated by many pathways, including β-adrenergic signalling which, when activated by β-receptor agonists, initiates the production of cAMP by *Adcy6*. This subsequently results in the phosphorylation of multiple downstream targets in cardiomyocytes, such as PLN, which collectively generate an increase in the frequency and force of contraction.^69^ Alterations in Ca^2+^ transporting or binding proteins can further attribute to hypertrophy, sarcomeric disorganization, and myofibrillar disarray, which affects the gross physiological state of the heart such as ventricular dilation and wall thinning.^50,70^

ADCY6 overexpression in mice has been previously linked to improved cardiac function due to increased cAMP production, resulting in increased phosphorylation of the Ca^2+^ regulatory protein PLN and subsequent activation of SERCA2a.^71–74^ This has been further confirmed in a pig HF model, in which intracoronary delivery of an adenovirus encoding ADCY6 resulted in improved LV function and prevented pathological remodelling due to increased cAMP production.^75^ Thus, the beneficial outcomes of ADCY6 in preclinical studies are promising and are currently under investigation in clinical trials on HF patients.^47,76,77^ In the current study, we observed an increase in phosphorylation of PLN in Zeb2-overexpressing hearts, indicating a relieve of its inhibitory effect on the Ca^2+^ pump SERCA2a, and consequently an increased Ca^2+^ uptake in the SR (*Figure 3*). Additionally, we observed that cardiomyocytes isolated from Zeb2 cTg mice post-IR displayed an increase in Ca^2+^ transient amplitude and a shorter decay time at higher frequencies, further supporting an improved Ca^2+^ reuptake in those cells (*Figure 5*). Our data suggest that the observed increase in PLN phosphorylation and improvement in Ca^2+^ handling occurs at least partially via the transcriptional regulation of *Adcy6* by ZEB2. This is supported by the presence of several conserved ZEB2 binding sites upstream of the transcriptional start site of ADCY6 and the upregulation of ADCY6 in cardiac tissue from ZEB2 Tg mice (see Supplementary material online, *Figure S4*). However, as RNA-seq indicated that ZEB2 overexpression in cardiomyocytes restored the expression of numerous genes related to Ca^2+^ homeostasis during ischaemic injury, additional mechanisms might at play to establish the cardioprotective effect.

An increase in intracellular Ca^2+^ concentration also results in the activation of calcineurin–NFAT signalling, which is one of the main drivers of pathological cardiac hypertrophy.^36,78^ Ca^2+^ overload in cardiomyocytes leads to activation of Ca^2+^/calmodulin-dependent phosphatase calcineurin. This results in the dephosphorylation of NFAT, its subsequent translocation to the nucleus, and activation of a hypertrophic gene programme.^36,37,78,79^ We observed increased levels of phosphorylated NFAT in ZEB2-overexpressing mice, which might be a direct consequence of improved Ca^2+^ handling. This reduction in calcineurin–NFAT signalling likely underlies the reduction in hypertrophy and maintained cardiac function in these hearts after ischaemic injury (*Figure 3*, Supplementary material online, *Figure S3*). Next to the herein described roles of ZEB2 in regulating Ca^2+^ homeostasis and myocyte function, we recently reported an additional cell non-autonomous function of ZEB2 in the injured heart.^22^ Genetic overexpression or AAV9-mediated delivery of ZEB2 to the injured heart triggered an increase in vessel density, diminished scar formation, and preserved cardiac function.^22^ Thus, ZEB2, on the one hand, contributes to creating a permissive environment for cardiac repair by enhancing angiogenesis and, on the other hand, controls the contractile function of cardiomyocytes, thereby preventing pathological remodelling.

Genetic deletion or therapeutic inhibition of *miR-208a* has been previously associated with enhanced cardiac function in a model of pressure overload in mice.^53,54^ In the context of ischaemic injury, we could show that anti-miR-mediated targeting of *miR-208a* increases ZEB2 expression and ameliorates cardiac hypertrophy and dysfunction (see Supplementary material online, *Figure S6*, and *Figure 6*). We delivered anti-208a to therapeutically increase Zeb2 levels in injured cardiomyocytes. However, this intervention did not fully recapitulate the protective effects we observed in the Zeb2 cTg mice post-IR as we did not observe an increase in phosphorylated NFATc3 levels after anti-208a treatment (*Figure 6*). MicroRNAs are modulators of gene expression and have multiple target genes.^80–82^ Based on target-predicting tools, miR-208a is predicated on regulating several phosphatases that could be involved in NFAT dephosphorylation which might explain our results. These observations are in line with our previous report in which we showed that *miR-208a* regulates the hypertrophic response in an NFAT-independent manner.^54^

Our study provides novel insights into the intricate molecular mechanisms governing cardiac responses to ischaemic injury. We demonstrate that ZEB2 is a key downstream regulator of HIF1α signalling, orchestrating cardiac Ca^2+^ handling and remodelling processes through transcriptional regulation of key genes like *Adcy6*. Additionally, our findings on the therapeutic targeting of miR-208a, which modulates ZEB2 expression, offer further avenues for exploring potential interventions to enhance cardiac function and mitigate hypertrophic responses in IHD (*Figure 7*). Subsequent investigations will focus on unveiling the downstream transcriptional network of ZEB2 to achieve a deeper molecular understanding of cardiac disease and cardioprotection, which will facilitate the development of targeted therapies.

**Translational perspective**

Our findings hold significant promise for advancing our understanding and treatment of ischaemia-induced HF and beyond. Intracellular calcium overload is a critical factor in cardiac dysfunction, yet effective therapies targeting this pathway remain elusive. Identifying ZEB2 as a regulator of contractility and calcium-handling components represents a vital step forward. By elucidating the molecular mechanisms underlying ZEB2’s protective effects, this research opens new avenues for therapeutic intervention. Targeting ZEB2 or its downstream pathways may offer novel strategies to mitigate HF progression and improve patient outcomes. Further translational and clinical studies are needed to validate these findings and assess the potential of ZEB2-targeted therapies in clinical settings.

## Supplementary Material

cvae163_Supplementary_Data

## Data Availability

Data are available from the corresponding author on reasonable request. RNA-seq data are deposited at the GEO repository under accession number GEO: GSE236827.
