# Supplementary material for: Hypoxia-responsive zinc finger E-box-binding homeobox 2 (ZEB2) regulates a network of calcium-handling genes in the injured heart
Source: Cardiovasc Res. 2024 Sep 23;120(15):1869–83. doi: 10.1093/cvr/cvae163 (PMC11630050; doi:10.1093/cvr/cvae163)
Supplement: cvae163_Supplementary_Data [file cvae163_supplementary_data.zip › Supplementary Figures.pdf]

Figure S1

a

Biological processes	Gene name
Homeostasis	*Anxa5*<br>*Anxa7*<br>*F2r*<br>*Entpd1*
Calcium-dependent cell-cell adhesion	*Pcdh18*<br>*Pcdhga9*
Regulation of apoptosis	*Htatip2*<br>*Madd*<br>*Rasa1*<br>*Adam17*<br>*F2r*<br>*Itm2b*<br>*Nr4a1*<br>*Serinc3*
Response to wounding	*Abhd2*<br>*Anxa5*<br>*F2r*<br>*Cfh*<br>*Mecom*<br>*Entpd1*
Positive regulation of transcription	*Htatip2*<br>*Egr1*<br>*Mecom*<br>*Nfia*<br>*Nfat5*<br>*Nr4a1*
Response to hypoxia	*Plat*<br>*Pdgfa*
Blood vessel development	*Htatip2*<br>*Rasa1*<br>*Pdgfa*<br>*Reck*
Proteolysis	*Fbxl5*<br>*Smurf2*<br>*Adam10*<br>*Adam17*<br>*Ctsc*<br>*Cfh*<br>*Hsp90b1*<br>*Nedd4*<br>*Plat*<br>*Rlim*<br>*Scpep1*
Extracellular matrix organization	*Smoc2*<br>*Col5a2*<br>*Reck*
Regulation of cell communication	*Adam17*<br>*Twsg1*<br>*Zeb2*

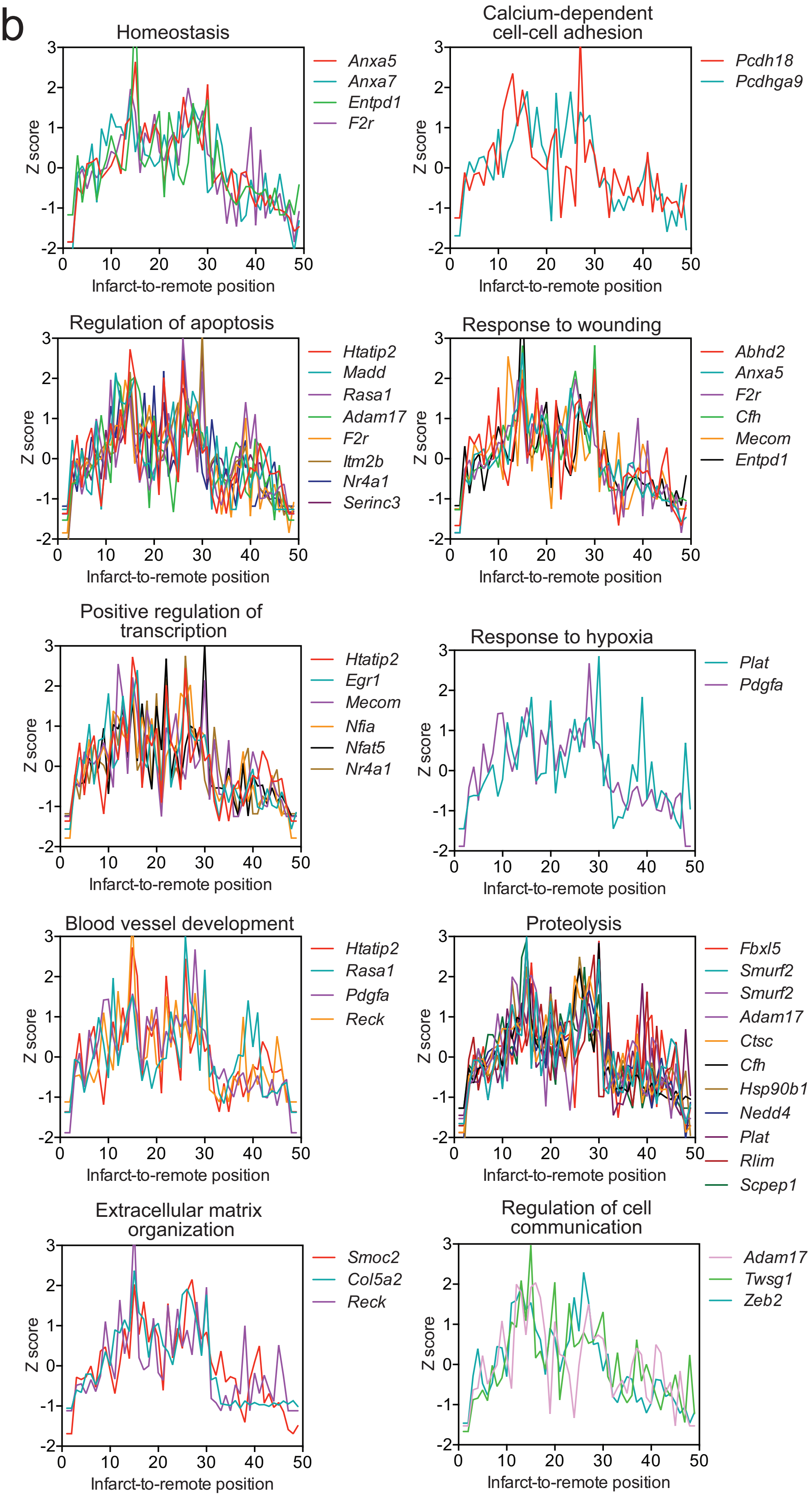

Supplementary Figure 1. Pathway enrichment and expression of *Hif1α* co-regulated genes. (a) Gene ontology analysis showing enriched biological processes (left) of *Hif1α* co-regulated genes (right). **(b)** Spatial expression traces of *Hif1α* co-regulated genes in mouse hearts 14 dpIR.

Figure S2

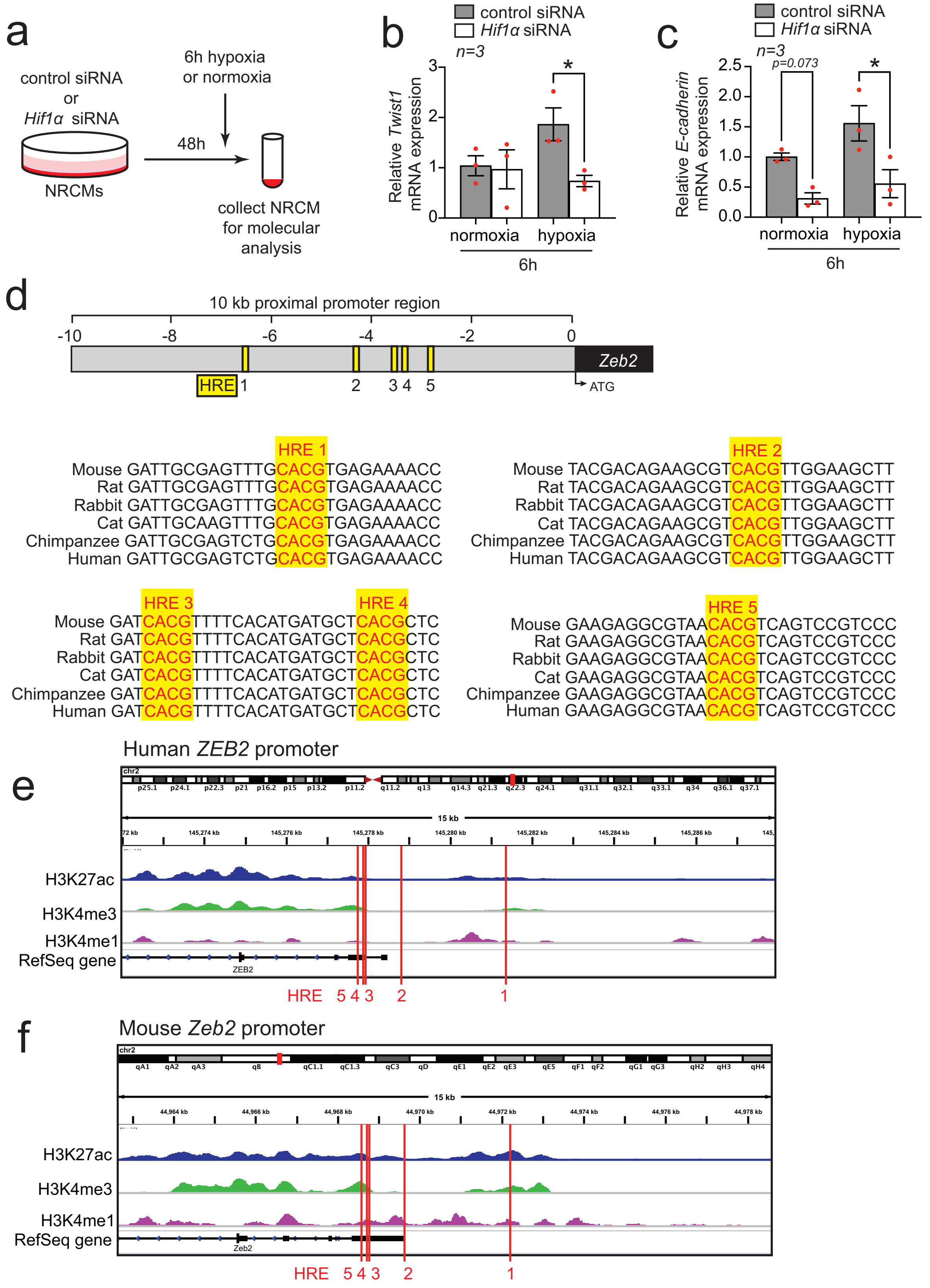

Supplementary Figure 2. *Hif1α* regulates *Zeb2* expression in cardiomyocytes. (a) Study design. **(b-c)** qPCR analysis of **(b)** *Twist1* and **(c)** *E-cadherin* expression levels following *Hif1α* knock down in NRCMs. **(d)** Upper panel: Schematic depicting the 10kb promoter region of *Zeb2* and multiple HRE; Lower panel: Sequences of *Zeb2* promoter regions showing conserved HRE among multiple species. **(e-f)** ChIP-seq profile of histone modifications in **(e)** human and **(f)** mouse *Zeb2* promoters. Red lines indicate conserved HRE. n (biological replicates) is indicated in the figures. Data are represented as mean ± SEM, *p < 0.05 compared to control using one-way ANOVA followed by Bonferroni's multiple comparison test **(b, c)**. NRCMs =Neonatal Rat Cardiomyocytes, HRE=Hypoxia Responsive Elements

Figure S3

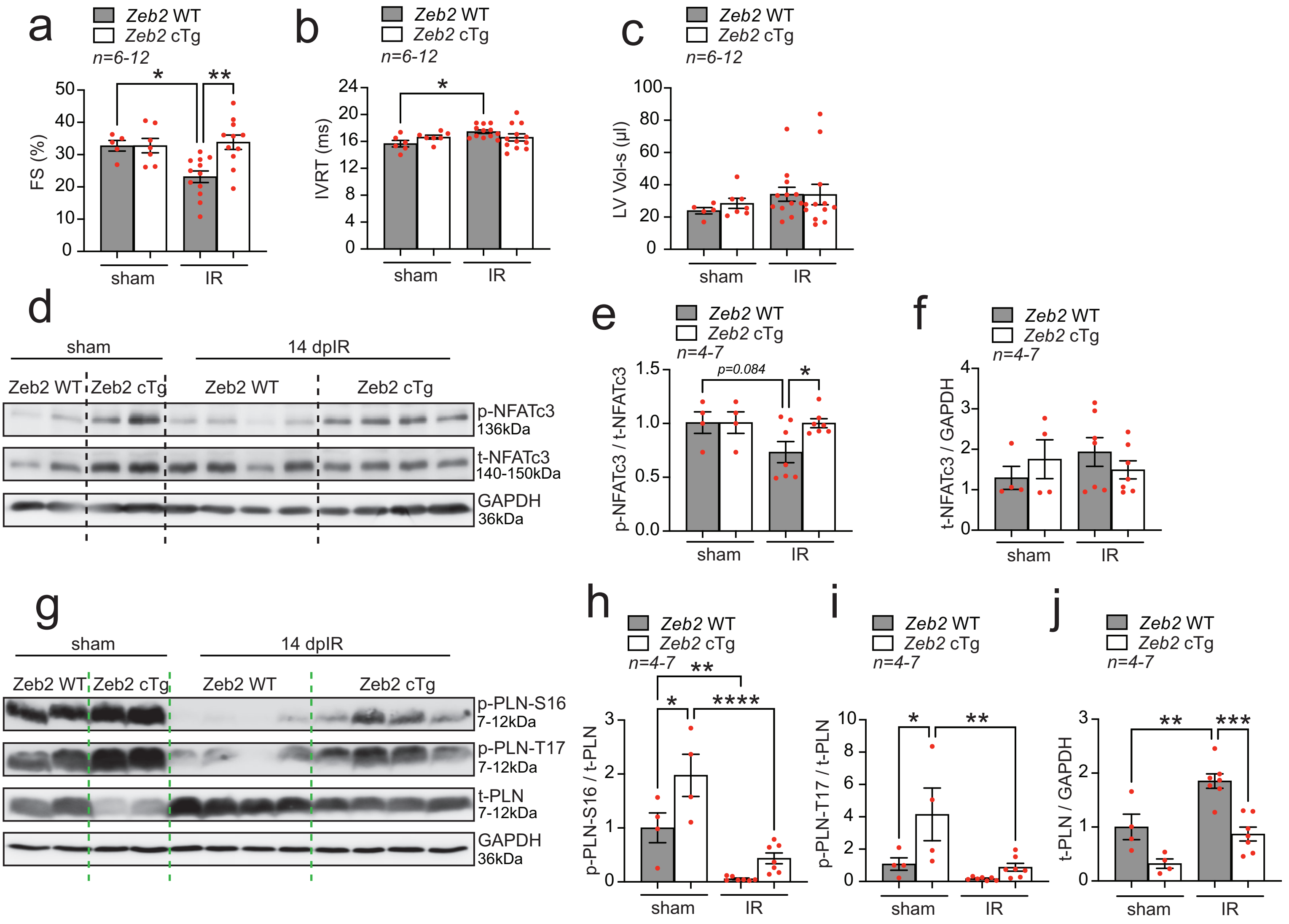

Supplementary Figure 3. Cardiomyocyte-specific ZEB2 overexpression in protects the heart from ischemic damage. **(a-c)** Quantification of **(a)** Fractional shortening (FS), **(b)** Isovolumic relaxation time (IVRT), **(c)** left ventricular volume in systole (LV Vol-s) in Zeb2 WT and Zeb2 cTg mice post sham or IR surgery. **(d-j)** Western blot analysis of **(d, g)** the indicated proteins and **(e-f, h-j)** their quantification in Zeb2 WT and Zeb2 cTg mice post-surgery. n (biological replicates) is indicated in figures. Data are represented as mean ± SEM, $*p < 0.05$, $**p < 0.01$, $***p < 0.001$, $****p < 0.0001$ using one-way ANOVA followed by Sidak multiple comparison test **(a, b, c, e, f, h, i, j)**

Figure S4

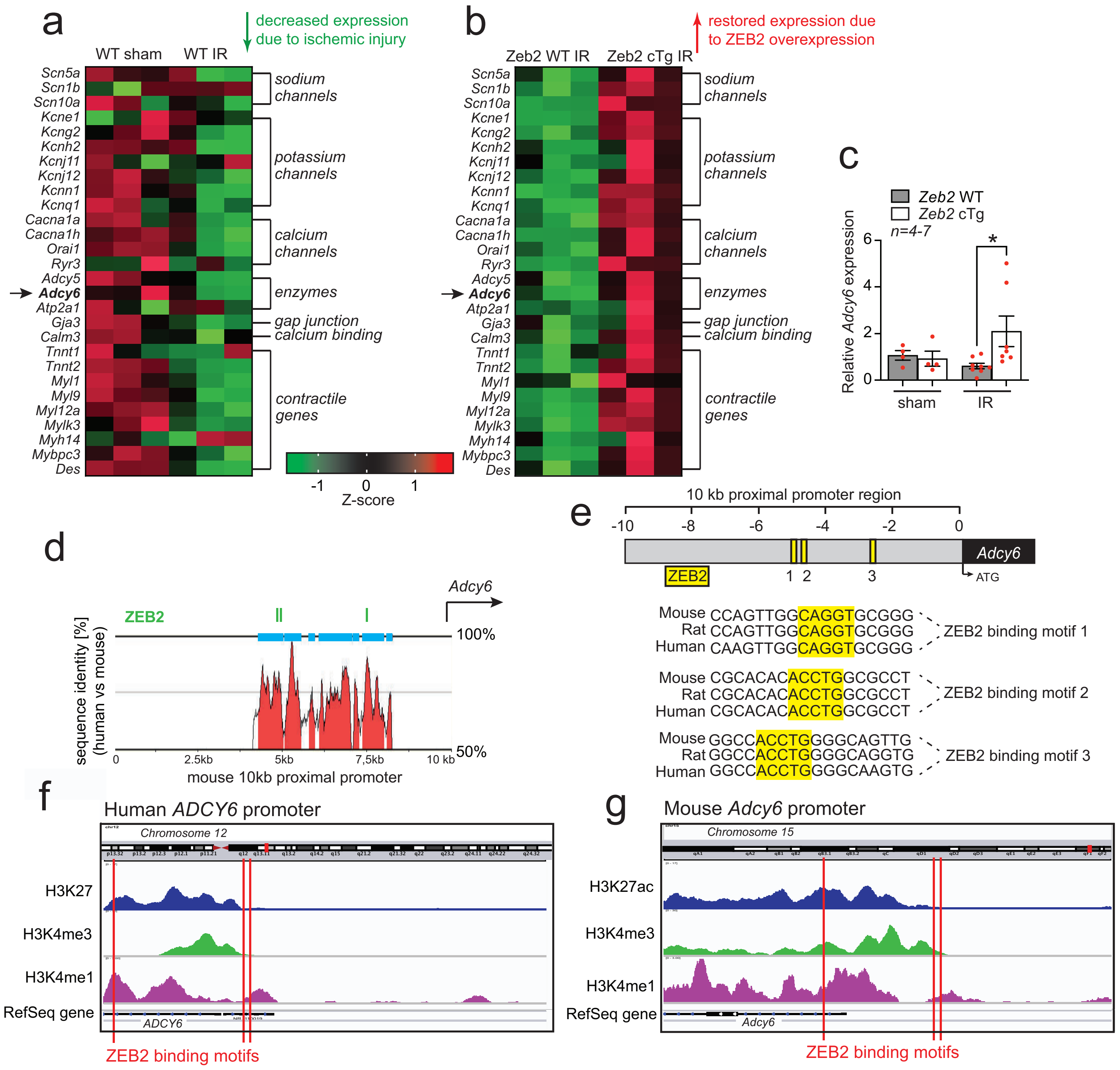

Supplementary Figure 4. ZEB2 overexpression restores the expression of contractile-related genes. (a-b) Heat maps showing the expression of contractile-related genes on RNA-seq from **(a)** WT sham versus WT IR and **(b)** Zeb2 cTg versus Zeb2 WT mice 14 dpIR. **(c)** qPCR analysis of *Adcy6* expression in Zeb2 WT and Zeb2 cTg mice post-surgery. **(d)** UCSC genome browser annotation of the 10kb proximal promoter region of *Adcy6* showing multiple conserved binding motifs for ZEB2. **(e)** Schematic depicting the 10kb promoter region of *Adcy6* and conserved sequences of multiple ZEB2-binding motifs. **(f-g)** ChIP-seq profile of histone modifications in human and mouse *Adcy6* promoters. **(h)** qPCR analysis of *Adcy6* expression following *Adcy6* knock down in normoxic and hypoxic NRCMs. (i) Western Blot analysis of ZEB2 expression and (j) the quantification following *Adcy6* knock down in normoxic and hypoxic cardiomyocytes NRCMs. Red lines indicate conserved ZEB2 binding motifs. n (biological replicates) is indicated in the figures. Data are represented as mean ± SEM, *p<0.05, **p<0.001 using one-way ANOVA followed by Sidak multiple comparison test **(c, h, j)**.

Figure S5

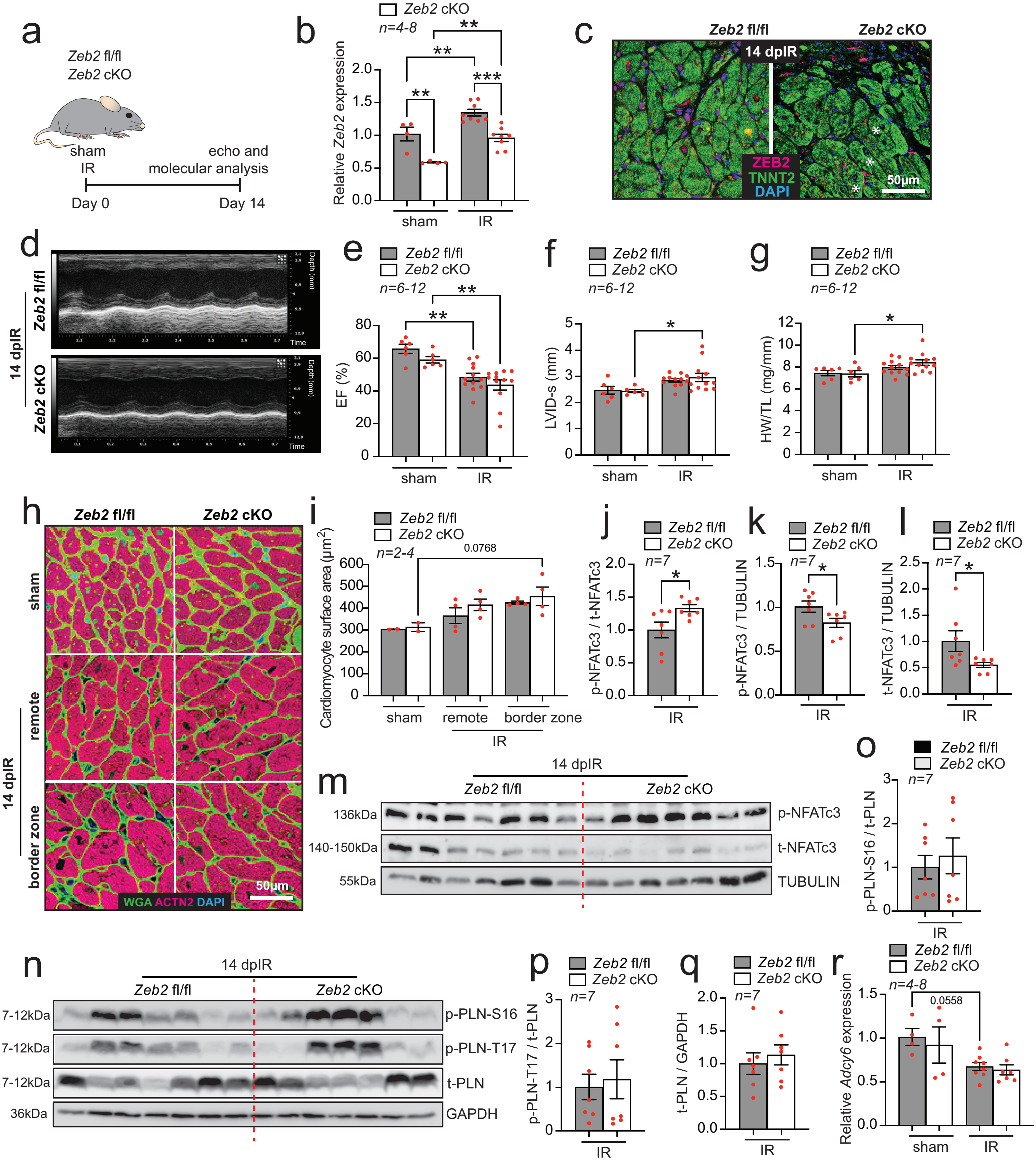

Supplementary Figure 5. Genetic deletion of ZEB2 from cardiomyocytes causes a mild decline of cardiac function post-ischemic injury but does not alter calcium signaling. (a) Study design. **(b)** qPCR analysis of *Zeb2* in hearts from Zeb2 fl/fl and Zeb2 cKO mice post-surgery. **(c)** Representative immunofluorescence staining of ZEB2 and TNNT2 in hearts from Zeb2 fl/fl and Zeb2 cKO mice 14 dpIR. **(d)** Representative M-mode images of hearts from Zeb2 fl/fl and Zeb2 cKO mice 14 dpIR. **(e-g)** Quantification of **(e)** ejection fraction (EF), **(f)** left ventricular internal diameter in systole (LVID-s), **(g)** heart weight to tibia length ratio (HW/TL) in Zeb2 fl/fl and Zeb2 cKO mice post-surgery. **(h)** WGA staining of cardiomyocytes and **(i)** surface area quantification. **(j-q)** Western blot analysis of **(m, n)** the indicated proteins and **(j-l, o-q)** their quantification in Zeb2 fl/fl and Zeb2 cKO mice 14 dpIR. **(r)** qPCR analysis of *Adcy6* expression in Zeb2 fl/fl and Zeb2 cKO mice 14 days post-surgery. n (biological replicates) is indicated in the figures. Data are represented as mean ± SEM, *p<0.05, **p<0.01, ***p<0.001 using one-way ANOVA followed by Sidak's multiple comparison test **(b, e, f, g, r)**, compared to sham using one-way ANOVA followed by Dunnett's multiple comparison test **(i)** or compared to Zeb2 WT using unpaired, two-tailed Student's t-test **(j, k, l, o, p, q)**.

Figure S6

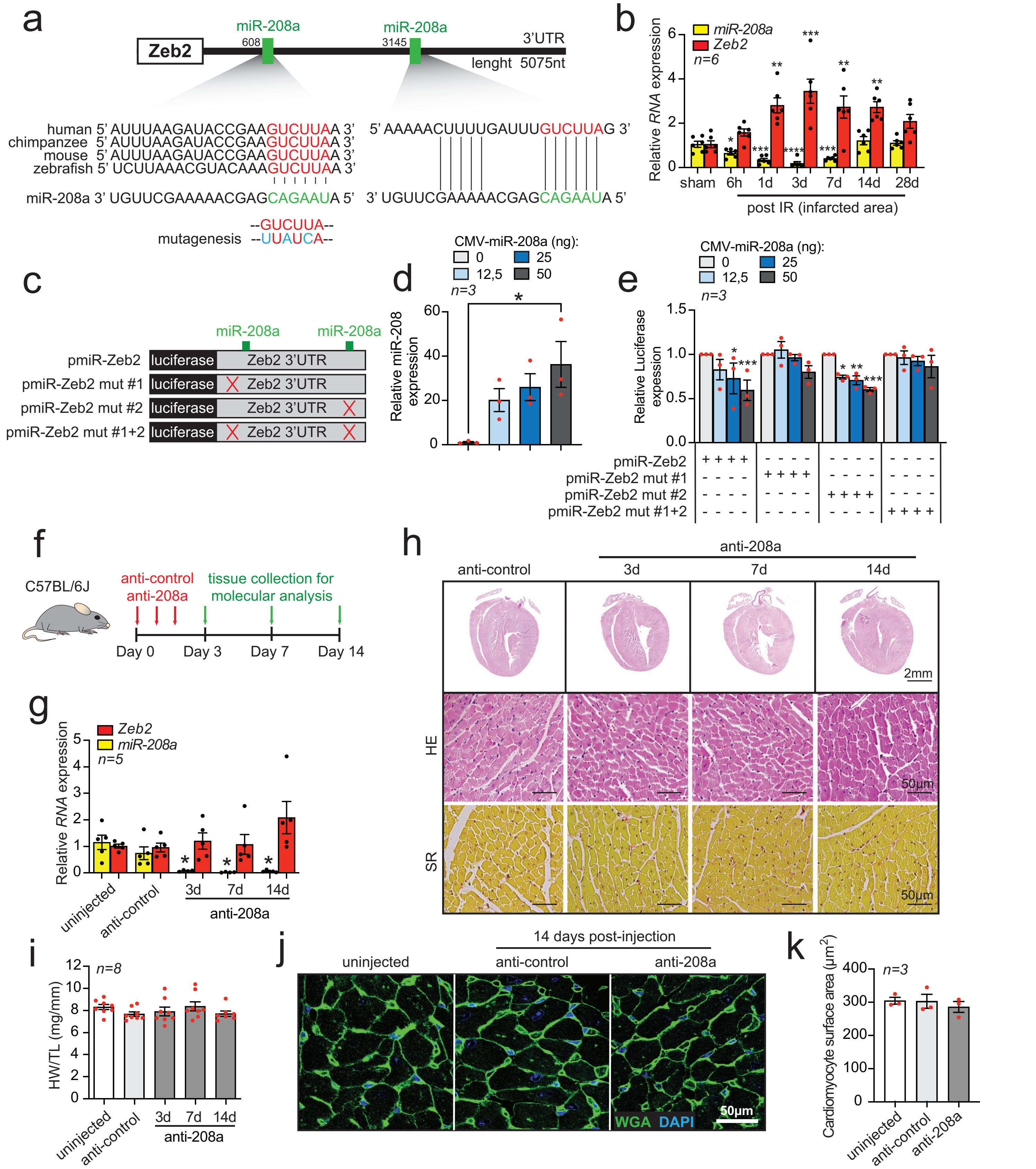

Supplementary Figure 6. *miR-208a* inhibits *Zeb2* expression in cardiomyocytes. (a) Schematic depicting the presence of 2 potential conserved binding sites for *miR-208a* in *Zeb2* 3'UTR and the designed mutated sites for luciferase reporter assay. **(b)** qPCR analysis of *Zeb2* and *miR-208a* expression levels in infarcted WT mouse heart tissue collected at different time points after IR surgery. **(c)** Schematic illustrating constructs designed for luciferase reporter assays containing WT and mutated *miR-208a* binding sites in *Zeb2* 3' UTR. **(d)** qPCR analysis of *miR-208a* expression levels following transfection of HEK-293 cells with different doses of CMV-miR-208a. **(e)** Luciferase reporter expression following transfection with WT and mutant constructs in **(c)**. **(f)** Study design. **(g)** qPCR analysis of *Zeb2* and *miR-208a* expression in WT mouse hearts treated with anti-208a. **(h)** Representative images of cardiac four-chamber view (top panels) and heart sections stained with H&E (middle panel) or SR (lower panels) following anti-208a treatment. **(i)** Quantification of HW/TL ratio of mice following anti-208a treatment. **(j)** WGA staining to show cardiomyocyte surface area and **(k)** its quantification. n (biological replicates) is indicated in the figures. HE, hematoxylin and eosin; SR, Sirius red. Data are represented as mean ± SEM, *p<0.05, **p<0.01, ***p<0.001 compared to corresponding control using one-way ANOVA followed by Dunnett's multiple comparison test **(b, d, g, I, k)** or two-way ANOVA followed by Bonferroni's multiple comparison test **(e)**.
