# Supplementary material for: Hypoxia-responsive zinc finger E-box-binding homeobox 2 (ZEB2) regulates a network of calcium-handling genes in the injured heart
Source: Cardiovasc Res. 2024 Sep 23;120(15):1869–83. doi: 10.1093/cvr/cvae163 (PMC11630050; doi:10.1093/cvr/cvae163)
Supplement: cvae163_Supplementary_Data [file cvae163_supplementary_data.zip › Zeb2 Supplementary material February2024.docx]

**Supplementary information**

**Hypoxia-responsive ZEB2 regulates a network of calcium handling genes in the injured heart.**

Monika M. Gladka^1,2^, Arwa Kohela^1,3^, Anne de Leeuw^1^, Bas Molenaar^1^, Danielle Versteeg^1^, Lieneke Kooijman^1^, Mariska van Geldorp^1^, Willem B. van Ham^4^, Rocco Caliandro^2^, Jody J. Haigh^5^, Toon A. B. van Veen^4^, Eva van Rooij^1,6 *^

^1^ Hubrecht Institute, Royal Netherlands Academy of Arts and Sciences (KNAW) and University Medical Centre Utrecht (UMCU), The Netherlands

^2^ Department of Medical Biology, Amsterdam University Medical Center, Amsterdam Cardiovascular Sciences, Amsterdam, The Netherlands

^3^ School of Biotechnology, Nile University, Giza, Egypt

^4^ Department of Medical Physiology, University Medical Centre Utrecht, The Netherlands

^5^ Department of Pharmacology and Therapeutics, University of Manitoba, Canada

^6^ Department of Cardiology, University Medical Centre Utrecht (UMCU), The Netherlands

**Supplementary information content:**

- **Methods**
- **Supplementary Figures with figure legends**
- **Supplementary Tables**

**METHODS**

**Mouse models.** Mice used in this study were generated as previously described. (1) Rosa26-LoxStopLoxZeb2 mice (2) were crossed with mice harbouring a Cre recombinase under the control of the murine *Myh6* promoter (αMHC-Cre Tg mice), (3) to generate αMHC-Cre R26-lslZeb2/lslZeb2 (*Zeb2* cTg) mice. Mice harbouring a floxed allele of *Zeb2* (*Zeb2* fl/fl*)* (4) were crossed to αMHC-Cre Tg mice to generate Cre-*Zeb2* fl/fl (*Zeb2* cKO) mice. Mouse studies were conducted in accordance with protocols approved by the ethics committee of the Hubrecht Institute, Utrecht, the Netherlands. Mice were housed in normal conditions with 12:12h light:dark cycles in a temperature-controlled room with food and water ad libitum. For all animal experiments, we used 8-9 weeks old male mice. All mice were genotyped by PCR using primers shown in **Supplementary Table 4**. The sample size was determined by a power calculation based on an echocardiographic effect size. Biotechnicians were blinded to group allocation during the experiment and when assessing the outcome.

**Ischemia-reperfusion.** Ischemia-reperfusion (IR) was performed by temporary (1 h) ligation of the left anterior descending artery (LAD). Mice were anaesthetized with a mixture of ketamine and xylazine by IP injection. Thorax area was shaved and cleaned with iodine and 70% ethanol. A tracheal tube was placed, and mice were connected to a ventilator (UNO Microventilator UMV-03, Uno BV.) Skin was subsequently incised at the midline to allow access to the left third intercostal space. Pectoral muscles were retracted, and the intercostal muscles were cut caudal to the third rib. Wound hooks were placed to allow access to the heart. The pericardium was incised longitudinally, and a 7.0 silk suture was placed around the LAD and a piece of 2-3 mm PE 10 tubing. One hour later, the PE tubing was removed, and the ligature was cut to allow for reperfusion. Following the surgery, mice were injected with 0.05-0.1mg/kg of buprenorphine (temgesic), the rib cage was closed with a 5.0 silk suture, and skin was closed with a wound clip. Mice were disconnected from the ventilator by removing the tracheal tube and placed on a nose cone with 100% oxygen. During the whole procedure and recovery period, animals were placed on a 38°C heating mat.

**Echocardiography.** Cardiac function was evaluated by two-dimensional transthoracic echocardiography on sedated mice (2% isoflurane) using a Visual Sonic Ultrasound system with a 30 MHz transducer (VisualSonics Inc., Toronto, Canada). Hearts were imaged in a parasternal long-axis and short-axis view at the level of the papillary muscles to record M-mode measurements and determine heart rate, wall thickness, and end-diastolic and end-systolic dimensions. Cardiac contractile function was assessed by Fractional Shortening (defined as the end-diastolic dimension minus the end-systolic dimension normalized for the end-diastolic dimension) and Ejection Fraction (defined as the stroke volume normalized for the end-diastolic volume).

**Tomo-seq.** Tomo-seq experiments were performed as described previously. (5) In short, heart samples were embedded in tissue freezing medium, frozen on dry ice, and cryosectioned into 48 slices of 80µm thickness. Next, RNA was extracted from individual slices and Illumina sequencing libraries were barcoded according to the CEL-seq protocol (Hashimshony et al., 2012). (6) Paired-end reads obtained by Illumina sequencing were aligned to the transcriptome using BWA (Li and Durbin, 2009). (7) The 5’ mate of each pair was used for mapping, discarding all reads that mapped equally well to multiple loci. The 3’ mate was used for barcode information. Reads counts were first normalized to total counts per section, and then renormalized to the median of total reads across sections in order to ensure that count numbers roughly corresponded to the number of mapped reads. Tomo-seq data analysis was performed in MATLAB (MathWorks) using custom-written code. An expression cut-off of >4 reads in >1 section was used.

**Isolation of ventricular cardiomyocytes from neonatal rats.** Neonatal rat cardiomyocytes (NRCM) were isolated by enzymatic dissociation of 1-2-day-old neonatal rat hearts. In brief, pups were placed on ice for 5-10 minutes for light anaesthesia. After decapitation, hearts were collected, ventricles were separated from atria and cut into small pieces in a balanced salt solution prior to enzymatic digestion using trypsin (Thermo Fisher Scientific, #15400054) under constant stirring at 37°C. The supernatant, containing intact cardiomyocytes was collected, centrifuged at 1500 rpm for 4 minutes and resuspended in Ham F10 medium (Thermo Fisher Scientific, #11550043) supplemented with 5% FBS, 10% L-glutamine and 10% Pen-Strep. Collected cells were seeded onto uncoated 100mm plastic dishes for 1,5 h at 37°C in 5% CO_2_ humidified atmosphere. Subsequently, the supernatant, which consists mainly of non-adhering CMs was collected, cells were counted and plated on gelatin-coated 6 well plates 1x10^6^ cells per well. After 24h medium was changed to Ham F10 supplemented with Insulin-Transferrin-Sodium Selenite Supplement (Roche), 10% L-glutamine and 10% Pen-Strep. Cells were used for the hypoxia study, siRNA-mediated knockdowns and infection with AAV9 virus as described below.

**Hypoxia study.** NRCMs were isolated and 1x10^6^ cells were plated per well in 6 well plates. For hypoxia treatment, cells were placed in a hypoxia chamber with 1% O_2_ and 5% CO_2_ and incubated for 1h, 2h, 4h, 6h, 8h or 24h. For normoxia treatment, control cells were placed for 24h in a regular cell culture incubator. RNA was isolated after each time point as described below. For immunohistochemistry, cells were cultured for 6h under normoxic or hypoxic conditions, fixed and stained as described below.

**Promoter analysis.** We used rVista 2.0 (https://rvista.dcode.org) to compare the *Zeb2* and *Adcy6* promoter regions between mouse and human and illustrated our data as percentage of conservation of a 10.0kb genomic region upstream of transcriptional start site of *Zeb2* or *Adcy6*.

**Measurements and analysis of calcium transients.** Cardiomyocytes were isolated as described before. (8) Cells were subsequently incubated in 1:1000 calcium-sensitive dye Fluo-4-AM (Thermofischer, #F14201) in Tyrode solution containing (mM): NaCl (130), KCl (4), CaCl_2_ (1.8), MgCl_2_ (1.2), NaHCO_3_ (18), HEPES (10), glucose (10), for 15 minutes at 37 degrees. Cells were placed in Tyrode solution during the recording of the calcium transients and were paced at 1Hz, 3Hz, and 5Hz by field stimulation. Recordings were made on a custom-built microscope (Cairn Research, Kent, UK) using a 10x objective. Blue light was used for excitation, using a 482/35 excitation filter (Semrock FF01-482/35-25), and captured using a 514 long-pass emission filter (Semrock LP02-514RU-25), with a high-speed camera (Andor Zyla 5.5.CL3, Oxford Instruments). Analysis was performed using a custom MATLAB script (https://osf.io/86ufe/).

**RNA isolation and quantitative real-time PCR (qPCR).** RNA was isolated using TRIzol reagent (Invitrogen) and reverse transcribed into cDNA using iScript cDNA Synthesis Kit (Bio-Rad, #1708891) according to the manufacturer’s instructions. qPCR was performed using iQ SYBR Green Supermix (Bio-Rad, #170-8885) on the CFX96 Realtime PCR instrument (Bio-Rad). Transcript levels were normalized for endogenous loading. Primer sequences are provided in **Supplementary Table 5**.

**RNA-sequencing.** Total RNA was extracted from remote zones of hearts using TRIzol reagent (Invitrogen). RNA sequencing libraries were prepared using the TruSeq Stranded Total RNA Library Prep Kit (Illumina) with Invitrogen according to the manufacturer’s instructions. Next, strand-specific single-end 75 bp reads were generated on an Illumina NextSeq 500. Reads were aligned and quantified against the Gencode.M4 gtf list for annotated genes using the STAR workflow. Heart libraries were sequenced with a minimum of 14 million reads (16.2 ± 1.9 (mean ± sd)). Differential expression was analyzed using DESeq v1.2211 using per condition dispersion estimates. *The sequencing data is currently being uploaded to the depository.*

**Gene ontology and pathway analysis.** To identify whether gene groups shared similar biological functions, differentially expressed gene groups were analysed using Kyoto Encyclopedia of Genes and Genomes (KEGG) pathway and gene ontology (GO) biological processes database using DAVID7. Significant enrichment of genes was shown, p values were corrected for multiple testing using the Benjamini-Hochberg method.

**Western blot analysis.** Heart tissue lysates were collected in RIPA buffer (50 mM Tris-HCl pH 7.5, 150mM NaCl, 0.1% SDS, 0.5% sodium deoxycholate (Sigma-Aldrich), 1% Triton X-100 (Sigma-Aldrich), protease inhibitor (Roche)), and protein concentration was determined using a Bradford assay (BioRad). Samples were boiled in 4x Leammli buffer, including 2% β- mercaptoethanol for 5 minutes at 99°C. SDS-PAGE and Western Blot were performed using Mini-PROTEAN Tetra Vertical Electrophoresis Cell with Mini Trans-Blot (Bio-Rad). Membranes were blocked in 3-5% non-fat dry milk and incubated overnight at 4°C with primary antibodies (**Supplementary Table 6**). On the next day, blots were incubated with the corresponding Peroxidase-conjugated AffiniPure secondary antibodies (Jackson ImmunoResearch) for 45 minutes and proteins were visualized using ECL solution (BioRad, #170-5061) on the ImageQuant LAS4000 imaging system. Western blots were quantified using Fiji software.

**Immunofluorescence staining.** Immunofluorescence imaging was performed on paraffin-embedded heart sections and fixed *in vitro* cultures. Paraffin-embedded heart sections were deparaffinized and re-hydrated in an alcohol gradient. Sections were subsequently boiled in boiling EDTA buffer pH 9 for 20 minutes for antigen retrieval, blocked with 0.05% BSA and incubated with primary antibodies (**Supplementary Table 5**) overnight at 4°C. On the next day, sections were washed and incubated with the corresponding Alexa Fluor secondary antibodies (Thermofisher Scientific) for 1 h followed by DAPI 1:5000 (Invitrogen, #D3571) for 10 minutes at room temperature (RT). Sections were finally mounted with ProLong Gold Antifade Mountant (Invitrogen, #P36934) for imaging. FITC-labeled wheat-germ-agglutinin (WGA) (Sigma-Aldrich, #L4895) was used to visualize and quantify cardiomyocyte cross-sectional area with ImageJ software. For *in vitro* cultures, cells were fixed with 4% PFA, quenched with NH4Cl, permeabilized, blocked with 1% fish gelatin (Gelatin from cold-water fish skin, Sigma-Aldrich, #G7765), and incubated with primary antibodies (**Supplementary Table 5**) for 25 minutes at RT. Cells were then incubated with the corresponding Alexa Fluor secondary antibodies (Thermofisher Scientific) for 20 minutes at RT. Cells were finally washed and sealed with mounting medium (ProLong Gold Antifade Mountant with DAPI, Invitrogen, #P36935). Imaging was performed using the Leica TCS SPE confocal microscope.

**siRNA experiments**

siRNA trilencers purchased from Origene were used to knock down ZEB2 (#SR511798) and HIF1α (#SR510711). A scrambled siRNA was used as a non-targeting control (#SR30002). Knock down was performed at a final concentration of 10nM using Lipofectamine 2000 (Thermo Fisher Scientific, #11668027) for 24h. Next, medium was refreshed for an additional 8h, and cells were harvested for analysis. Cells were subjected to hypoxia (1% O2 and 5% CO2) for 6 h before collection.

**Human heart samples.** Approval for studies on human tissue samples was obtained from the Medical Ethics Committee of the University Medical Center Utrecht, The Netherlands (12#387). Written informed consent was obtained or in certain cases waived by the ethics committee when obtaining informed consent was not possible due to death of the patient. In this study, we used tissue from the left ventricular free wall of patients with end-stage heart failure secondary to ischemic heart disease. Tissue was obtained during heart transplantation or upon autopsy. RNA was isolated as previously described and gene expression values obtained by qPCR were plotted for correlation analysis.

**MicroRNA target prediction.** For the identification of putative microRNAs targeting Zeb2, we used the target prediction tools miRBase (http://www.mirbase.org) and TargetScan (http://www.targetscan.org/vert_72/).

**Luciferase assay.** HEK293T cells were transfected using Lipofectamine (Thermo Fisher Scientific, #11668027) with pMIR-reporter plasmid containing the 3’UTR of ZEB2 (25 ng/well), pCMV plasmid containing miR-208a (at different concentrations) and renilla. After 48 hours luciferase activity was measured using the Dual-Luciferase® Reporter Assay System (Promega). Relative luciferase activity was normalized to renilla expression.

**Anti-miR injection in mice**

For the baseline study, adult C56BL/6J mice (Charles Rivers) were injected with antimiR-control or antimiR-208a (25mg/kg) subcutaneously for three consecutive days. Animals were sacrificed 3, 7 or 14 days after the last injection and cardiac tissues were collected for baseline analysis. For the IR study, adult mice received sham or IR surgery which was followed by subcutaneous antimiR injections for three consecutive days and used for functional and molecular analysis 14 days later.

**Statistical and reproducibility.** The number of samples (n) used in each experiment is shown in the figures and indicates biological replicates. Results are presented as the mean ± standard error of the mean (SEM). Statistical analyses were performed using PRISM (GraphPad Software Inc. version 6). Two groups were statistically compared using Student’s *t*-test. Multiple groups were statistically compared using ordinary one-way ANOVA or two-way ANOVA. Outliers were defined by Grubbs’ test (alpha=0.05). Data are represented as mean ± SEM. Differences were considered statistically significant at p<0.05. In the figures, asterisks indicate statistical significance (*p<0.05, **p<0.01, ***p<0.001, ****p<0.0001) which is also indicated in the individual figures. All representative images of hearts or cells were selected from at least three independent experiments with similar results, unless indicated differently in the figure legend.

**Supplementary Table 4. Sequences of genotyping primers.**

| **Gene name** | **Forward primer** | **Reverse primer** |
| --- | --- | --- |
| *Cre* | gaagcaactcatcgattgatttacg | cactatccaggttacggatatagttc |
| *Zeb2* floxed allele | gaactagttgaattggtagaatcaatgggg (intron 6) | atcagcagcctcctatttaaacagagtgtc (intron 6)  aagcatgtcggtaagctgaccaactactag (intron 7) |
| *Zeb2* knock in | aaagtcgctctgagttgttat | ggagcgggagaaatggatatg (wild type)  gcgaagagtttgtcctcaacc (knock in) |

**Supplementary Table 5. Sequences qPCR primers.**

| **Gene name** | **Species** | **Forward primer** | **Reverse primer** |
| --- | --- | --- | --- |
| *Adcy6* | Mouse | ctggaggatgagaatgagaagc | tgcctctggatgtaaatcttgtg |
| *Cacna1a* | Mouse | ggtgagaaaatacgccaaaaag | atgggtgtcttgtcatcatcag |
| *Cox4i1* | Mouse | ttccagggatgagaaagttcag | tcttctcccaaatcagaacgag |
| *Cox8b* | Mouse | aagttcacagtggttcccaaag | atagctctccaagtgggctaag |
| *E-cadherin* | Rat | tgaagcccaggaaatacacc | caccaacacacccagcatag |
| *eGFP* |  | acgacttcttcaagtccgcc | tcttgtagttgccgtcgtcc |
| *Gapdh* | Mouse | tgtcgtggagtctactggtg | acacccatcacaaacatgg |
| *HIF1A* | Human | aggcttaccatcagctatttgc | accataacaaaaccatccaagg |
| *Hif1a* | Rat | acaaagctcacctgagcctaac | gtcctgagctgaaaatggattc |
| *Hif1a* | Mouse | cccacaatgtgagctcacatct | ccatctgtgccttcatctcatctt |
| *Kcnj12* | Mouse | cctgcagatgccaggacaag | cagctggcctgaactagacaa |
| *miR-208a* | Mouse | ataagacgagcaaaaagcttgt | gaatcgagcaccagttacg (universal) |
| *Nppa* | Mouse | ggtaggattgacaggattggag | gcttaggatcttttgcgatctg |
| *Nppb* | Mouse | gagtccttcggtctcaaggc | caacttcagtgcgttacagc |
| *Nt5e* | Mouse | gtttacaaaggccttgaagtgg | ggatcaatcagtccttccacac |
| *Tnni1* | Mouse | ctgagaaggtgcgttacctc | tggaggcatttggcttcaat |
| *Twist1* | Rat | gaggagctgcagacacagc | gcaggacctggtacaggaag |
| *ZEB2* | Human | gacattccagaaaagcagttcc | gaagccttgagtgctcgataag |
| *Zeb2* | Rat | ccgttggacctgtcattacc | ggatgaagaaacactgttgtgg |
| *Zeb2* | Mouse | gagcaggtaaccgcaagttc | aagcgtttcttgcagtttgg |

**Supplementary Table 6. List of antibodies.**

| **Antibody** | **Company** | **Catalogue nr.** | **Purpose and dilution** |
| --- | --- | --- | --- |
| ACTN2 | Sigma-Aldrich | A7732 | Immunostaining 1:1000 |
| TNNT2 | Abcam | ab8295 | Immunostaining 1:800  Western blot 1:1000 |
| ZEB2 | Novus- biologicals | NBP1-77179 | Immunostaining 1:250 |
| p-NFATc3 | Santa Cruz Biotechnology | sc-365786 | Western blot 1:200 |
| t-NFATc3 | Santa Cruz Biotechnology | sc-8405 | Western blot 1:200 |
| GAPDH | Millipore | MAB374 | Western blot 1:5000 |
| p-PLN-S16 | Badrilla | A010-12AP | Western blot 1:1000 |
| p-PLN-T17 | Badrilla | A010-13AP | Western blot 1:1000 |
| t-PLN | Thermo Fisher Scientific | MA3-922 | Western blot 1:1000 |
| TUBULIN | Sigma-Aldrich | T9026 | Western blot 1:1000 |

**References**

1. Gladka MM, Kohela A, Molenaar B, Versteeg D, Kooijman L, Monshouwer-Kloots J, et al. Cardiomyocytes stimulate angiogenesis after ischemic injury in a ZEB2-dependent manner. *Nat Commun.* 2021;12(1):84.

2. Nyabi O, Naessens M, Haigh K, Gembarska A, Goossens S, Maetens M, et al. Efficient mouse transgenesis using Gateway-compatible ROSA26 locus targeting vectors and F1 hybrid ES cells. *Nucleic Acids Res.* 2009;37(7):e55.

3. Agah R, Frenkel PA, French BA, Michael LH, Overbeek PA, and Schneider MD. Gene recombination in postmitotic cells. Targeted expression of Cre recombinase provokes cardiac-restricted, site-specific rearrangement in adult ventricular muscle in vivo. *J Clin Invest.* 1997;100(1):169-79.

4. Higashi Y, Maruhashi M, Nelles L, Van de Putte T, Verschueren K, Miyoshi T, et al. Generation of the floxed allele of the SIP1 (Smad-interacting protein 1) gene for Cre-mediated conditional knockout in the mouse. *Genesis.* 2002;32(2):82-4.

5. Lacraz GPA, Junker JP, Gladka MM, Molenaar B, Scholman KT, Vigil-Garcia M, et al. Tomo-Seq Identifies SOX9 as a Key Regulator of Cardiac Fibrosis During Ischemic Injury. *Circulation.* 2017;136(15):1396-409.

6. Hashimshony T, Wagner F, Sher N, and Yanai I. CEL-Seq: single-cell RNA-Seq by multiplexed linear amplification. *Cell Rep.* 2012;2(3):666-73.

7. Li H, and Durbin R. Fast and accurate short read alignment with Burrows-Wheeler transform. *Bioinformatics.* 2009;25(14):1754-60.

8. Louch WE, Sheehan KA, and Wolska BM. Methods in cardiomyocyte isolation, culture, and gene transfer. *J Mol Cell Cardiol.* 2011;51(3):288-98.
